# WNT signaling modulates PD-L1 expression in the stem cell compartment of triple-negative breast cancer

**DOI:** 10.1038/s41388-019-0700-2

**Published:** 2019-01-31

**Authors:** Lorenzo Castagnoli, Valeria Cancila, Sandra L. Cordoba-Romero, Simona Faraci, Giovanna Talarico, Beatrice Belmonte, Marilena V. Iorio, Matteo Milani, Tatiana Volpari, Claudia Chiodoni, Alfredo Hidalgo-Miranda, Elda Tagliabue, Claudio Tripodo, Sabina Sangaletti, Massimo Di Nicola, Serenella M. Pupa

**Affiliations:** 1 Molecular Targeting Unit, Department of Research, Fondazione IRCCS Istituto Nazionale dei Tumori di Milano, Milan, Italy; 2 Tumor Immunology Unit, Department of Health Science, Human Pathology Section, University of Palermo School of Medicine, Palermo, Italy; 3 Molecular Immunology Unit, Department of Research, Fondazione IRCCS Istituto Nazionale dei Tumori di Milano, Milan, Italy; 4 Unit of Immunotherapy and Anticancer Innovative Therapeutics, Department of Medical Oncology and Hematology, Fondazione IRCCS Istituto Nazionale dei Tumori di Milano, Milan, Italy; 5 Cancer Genomics Laboratory, National Institute of Genomic Medicine, Mexico City, Mexico

**Keywords:** Cell biology, Stem cells, Immunology

## Abstract

Triple-negative breast cancers (TNBCs) are characterized by a poor prognosis and lack of targeted treatments, and thus, new therapeutic strategies are urgently needed. Inhibitors against programmed death-1 (PD-1)/PD-1 ligand (PD-L1) have shown significant efficacy in various solid cancers, but their activity against TNBCs remains limited. Here, we report that human TNBCs molecularly stratified for high levels of PD-L1 (PD-L1^High^) showed significantly enriched expression of immune and cancer stemness pathways compared with those with low PD-L1 expression (PD-L1^Low^). In addition, the PD-L1^High^ cases were significantly associated with a high stemness score (SS^High^) signature. TNBC cell lines gated for aldehyde dehydrogenase (ALDH) and CD44 stemness markers exhibited increased levels of PD-L1 *versus* their ALDH-negative and CD44^Low^ counterparts, and PD-L1^High^ cells generated significantly more mammospheres than PD-L1^Low^ cells. Murine mammary SCA-1-positive tumor cells with PD-L1^High^ expression generated tumors in vivo with higher efficacy than PD-L1^Low^ cells. Furthermore, treatment of TNBC cells with selective WNT inhibitors or activators downregulated or upregulated PD-L1 expression, respectively, implying a functional cross-talk between WNT activity and PD-L1 expression. Remarkably, human TNBC samples contained tumor elements co-expressing PD-L1 with ALDH1A1 and/or CD44v6. Additionally, both PD-L1-/SCA1-positive and ALDH1A1-positive tumor elements were found in close contact with CD3-, and PD-1-positive T cells in murine and human tumor samples. Overall, our study suggests that PD-L1-positive tumor elements with a stemness phenotype may participate in the complex dynamics of TNBC-related immune evasion, which might be targeted through WNT signaling inhibition.

## Introduction

Breast cancer (BC) is the most common malignant disease and the second-leading cause of cancer-related death in women worldwide [1]. Triple-negative breast cancer (TNBC) is a particularly aggressive molecular BC subtype characterized by the lack of expression of estrogen and progesterone receptors and HER2 oncoprotein, the three “gold standard” actionable biotargets for luminal and HER2-enriched BCs [2]. Currently, due to their vast inter- and intra-tumor molecular heterogeneity, TNBCs have no specific therapeutic targets, which urgently need to be identified [2]. Recent advances in gene expression profiling have identified distinct molecular TNBC subsets that, if appropriately selected, might be more responsive to tailored therapies with available agents [2, 3]. Although the majority of TNBC presents a consistent intra-tumor immune infiltrate, which has been shown to have prognostic significance [4–6], the mechanisms by which such tumors evade anti-tumor immune attack are still unclear. Binding of programmed death-1 (PD-1) protein, an inhibitory immune checkpoint receptor expressed on activated T cells, to the immunosuppressive signal PD-1 ligand (PD-L1) expressed either on cancer cells or immunosuppressive cells in the tumor microenvironment, represents one of the driving mechanisms of tumor immune escape [7–9]. The clinical availability of immune checkpoint inhibitors able to block the PD**-**1/PD-L1 axis has revolutionized the therapeutic scenario of different advanced cancers [10], and recently, different investigations in advanced TNBC have led to preliminary evidence of a modest clinical activity of both PD-1 and PD-L1 inhibitors used as monotherapy or in combination with chemotherapy [11–13]. Specifically, using TCGA data [14], it has been shown that TNBCs express significantly higher PD-L1 transcript levels than non-TNBC tissues [10, 15], and, in addition, the response rates to immunotherapy appear to be higher in cases with PD-L1 expression in tumor cells [8]. However, the relative roles and functions of PD-L1 on tumor cells and on other immune cell types in the tumor microenvironment remain elusive, and their elucidation is pivotal to understanding and predicting immunotherapeutic success or failure in BCs [16].

Emerging data underline that the clinical benefits mediated by molecular targeted therapies are related to their ability to efficiently target cancer stem cells (CSCs), a small subset of malignant cells with unlimited self-renewal capacity that are mainly responsible for tumor growth, progression, metastasis and resistance to chemo and radiotherapies, targeted agents, anti-angiogenics, and immunotherapies, all of which are features linked to a poor clinical outcome [17]. Therefore, understanding CSC regulation and maintenance appears to be a high priority in the attempt to achieve long-lasting tumor control/eradication and eventually achieve a cure [17]. Very recent evidence has underlined the involvement of CSCs in the negative modulation of immune responses in the early phases of carcinogenesis through different biological mechanisms [18, 19]. In particular, a close relationship between the upregulation of tumor-restricted PD-L1 and human CSC markers, basal cell markers and vimentin expression has been reported in invasive BC [20], as well as constitutive and inducible expression of PD-L1 in the CD44-positive CSC subset of head and neck squamous cell carcinoma [21]. Overall, these findings lead to the speculation that PD-L1 can work as a molecular “shield” to protect CSCs from T cell lysis.

In this study, we show that TNBC stem cells (TNBCSCs) constitutively upregulate PD-L1 through the activity of the WNT signaling pathway, the inhibition or activation of which significantly affects PD-L1 expression. Moreover, we provide in vivo evidence of the close contact between tumor PD-L1-positive elements with the stemness phenotype and CD3-, and PD-1-expressing infiltrating T cells. Overall, our findings demonstrate an in vivo TNBC-related scenario that could potentially be reverted through the use of WNT inhibitors, which are already in phase 1 clinical trials [22], with the aim of downregulating PD-L1 expression to restore an effective anti-tumor immune response.

## Results

### High PD-L1 expression (PD-L1^High^) in human TNBCs is significantly associated with stem-like- and immune-related features

**Fig. 1:**
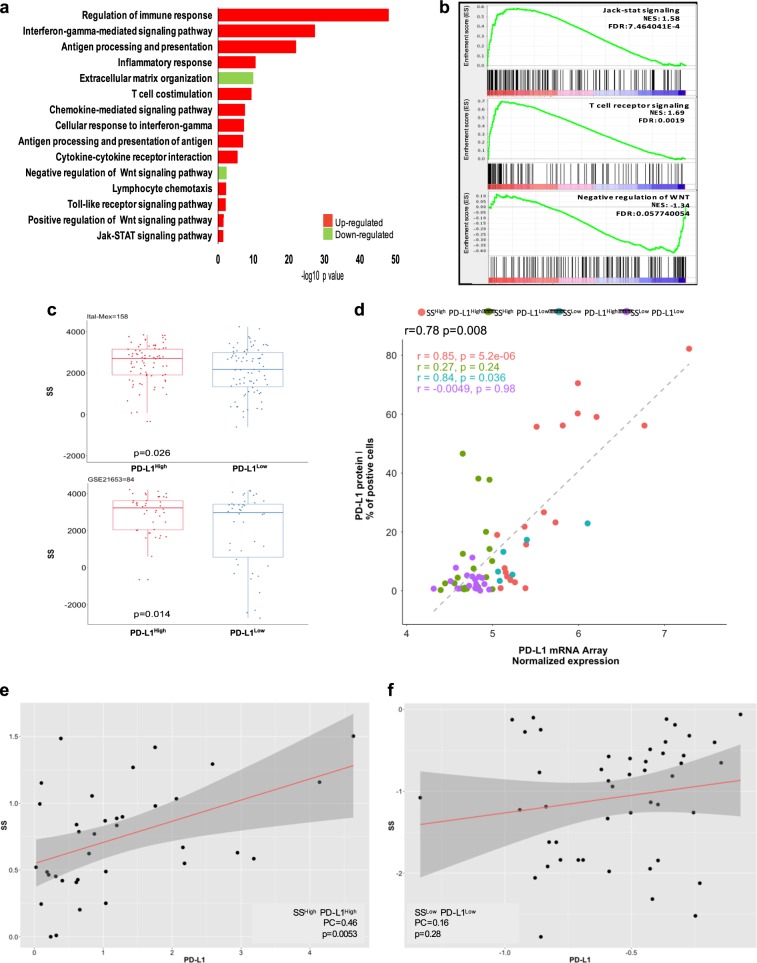
In silico analyses of the Ital-Mex TNBC cohort and GSE21653 stratified according to PD-L1 expression. **a** KEGG analysis of the gene pathways differentially expressed in PD-L1^High^ and PD-L1^Low^ TNBC cases (tumors divided by median, *n* = 158). Bar plot shows significantly enriched gene pathways upregulated in PD-L1^High^ Ital-Mex TNBC cases *versus* PD-L1^Low^. The bar plot shows the significant top enrichment scores (−log *p* value). **b** GSEA enrichment plots of Jak-stat signaling, T cell receptor signaling, and negative regulation of WNT gene sets in PD-L1^High^ compared with PD-L1^Low^ TNBC cases. The enrichment score (ES) describes the degree to which a gene set is overrepresented in the ranked list of genes. The NES computes the density of modified genes by the number of genes annotated in each gene cluster, allowing comparisons between conditions. In every panel, the green curve represents the running ES for the gene set as the analysis moves down in the ranked list. The maximum peak is the final ES computed for the gene set (peak score). The middle portion of the plot (lines representation) shows where the gene members of the gene set appear in the ranked list and the expression status described by the color heat-map (red, over-expressed; blue, down-modulated). The leading-edge subset, which represents the gene members that contributed most to the ES, is shown as follows: for a positive ES, the leading edge appears to the left of the maximum peak (left side of the plot), and for a negative ES, the leading edge appears subsequent to the peak score (right side of the plot). **c** Upper panel: boxplot showing the distribution of SS in PD-L1^High^ and PD-L1^Low^ TNBC cases (cutoff median) of the Ital-Mex cohort, and **c** lower panel: GSE21653 validation cohort (*n* = 84). **d** Scatter plot of PD-L1 expression levels evaluated by IHC (*y*-axis) and mRNA array (*x*-axis) in 63 matched TNBC cases stratified as SS^High^/PD-L1^High^ (*n* = 19), SS^High^/PD-L1^Low^ (*n* = 20), SS^Low^/PD-L1^High^ (*n* = 6), SS^Low^/PD-L1^Low^ (*n* = 18). **e** Scatter plot of PD-L1 expression levels (*z*-score, *x*-axis) and stemness score (*z*-score, *y*-axis) in ITAL-MEX TNBC cases sub-classified for SS^High^/PD-L1^High^ (*n* = 36) or **f** for SS^Low^/PD-L1^Low^ (*n* = 40). The correlation status was calculated by a Pearson correlation and the *p*-value for each of the two tumor groups

To better characterize the transcriptional landscape of PD-L1-positive TNBCs, we analyzed genome-wide RNA expression profiles in a cohort of 158 cases (Ital-Mex) processed in house. Enrichment pathway analysis revealed the following: (1) the up-modulation of distinct immune-related signaling; (2) upregulation of positive WNT signaling regulation (pathways involved in the activation or increment of Wnt signaling) and consequent loss of negative WNT signaling regulation (pathways involved in the arrest or prevention of Wnt signaling) [23, 24]; and (3) over-representation of Jak-STAT signaling pathways among the cellular processes most significantly enriched in PD-L1^High^ tumors (Fig. 1a). Similar results were obtained by gene set enrichment analysis (GSEA) [25], showing a strong significant enrichment of T cell receptor signaling (normalized enrichment score (NES) 1.69), Jack-STAT signaling (NES 1.58) and the down-representation of negative regulation of WNT in PD-L1^High^ TNBCs (NES **−**1.34) (Fig. 1b). The significant differential expression of WNT pathway-related genes observed in PD-L1^High^ TNBCs *versus* those expressing low levels (PD-L1^Low^) strongly suggests that PD-L1 can play a biological role in the stemness of this BC subtype. To evaluate the association of an enhanced stem-like phenotype with PD-L1^High^ levels, we reviewed the Ital-Mex dataset with the already reported stemness score (SS) signature [26]. As shown in Fig. 1c (upper panel), PD-L1^High^ TNBCs from the Ital-Mex cohort showed a significantly higher SS than PD-L1^Low^ samples (*p* = 0.026). Additionally, an independent evaluation of a public dataset (GSE21653, *n* = 84) confirmed our results (Fig. 1c, lower panel). Since the Immunohistochemical staining of PD-L1 in tumors cells has been used as a gold standard to detect PD-L1 positivity (Supplementary Fig S1), we compared PD-L1 mRNA expression with its protein levels in the Ital-Mex cohort. In particular, PD-L1 protein (immunofluorescence) and mRNA (microarray) expression were evaluated in 63 matched tumors, where they resulted significantly correlated (Pearson = 0.78, *p* = 0.0008) (Fig. 1d). In particular, PD-L1^High^ tumors, classified by gene expression profile, revealed the most robust correlation (SS^High^/PD-L1^High^ Pearson = *p* = 0.85, *p* = 0.00005, SS^High^/PD-L1^Low^ Pearson = 0.84, *p* = 0.036; Fig. 1d). To further explore the potential correlation of stem-like phenotype and increased PD-L1 levels in human TNBC cases, we sub-classified our cohort in groups according to their PD-L1 and SS expression levels as follows: SS^High^/PD-L1^High^, *n* = 36; SS^Low^/PD-L1^Low^, *n* = 40; SS^High^/PD-L1^Low^, *n* = 55; and SS^Low^/PD-L1^High^, *n* = 20. According to our previous findings, SS^High^/PD-L1^High^ TNBCs presented a positive correlation between SS and PD-L1 expression (Pearson = 0.46, *p* = 0.005), while SS^Low^/PD-L1^Low^ tumors did not present any significant correlation (Pearson = 0.16, *p* = 0.28) (Fig. 1e, f). Additionally, SS^High^/PD-L1^Low^ (Pearson = −0.29, *p* = 0.029) and SS^Low^/PD-L1^High^ (Pearson = 0.22, *p* = 0.004) tumors showed moderate relationships between SS and PD-L1 expression levels, indicating weak relationships between these two parameters (Supplementary Fig. S2). Notably, PD-L1^High^ TNBCs also presented a significantly higher claudin-low score (Supplementary Fig. S3a), a tumor phenotype known to reflect an enrichment of stem-like/epithelial mesenchymal transition (EMT) features [27]. To address the potential contribution of stromal cell compartments to PD-L1 expression pattern across TNBC, we computed ESTIMATE algorithm [28] to infer the fraction of stromal and immune cells in the bulk gene expression profiles. A global median purity of 67% was calculated among Ital-Mex cohort, in accordance to our sample inclusion criteria. Tissue samples showed heterogeneous levels of tumor purity, with SS^High^/PD-L1^High^ and SS^High^/PD-L1^Low^ tumors being the most enriched in cancer cells (Supplementary Fig. S3b). Moreover, SS^Low^/PD-L1^Low^ showed a significantly higher stromal infiltration compared to the other tumor clusters (Supplementary Fig. S3c). As expected, both SS^High^/PD-L1^High^ and SS^Low^/PD-L1^High^ subgroups showed a significant increase in the immune cell enrichment (Supplementary Fig. S3d). We then used a complementary strategy to explore the immune content through the Immunophenoscore (IPS), a transcriptional-based score [29]. PD-L1^High^ subgroups were indeed the more immunogenic phenotypes (Supplementary Fig. S3e). Overall, SS^High^/PD-L1^High^ samples demonstrate a considerable restricted stromal infiltration, significant immunological enrichment and high tumor cell content. The meaning of enriched tumor purity based on genomic measurements and pathological evaluation indicates that high tumor content increases the sensitivity of tumor transcriptomic alteration detection. Thus, we conclude that both bulk and CSC subpopulations are the major contributors of the PD-L1 expression patterns in the evaluated cohort. Overall, these data provide clear evidence of the significant enrichment of immune-related and stem-like WNT signaling gene pathways in human TNBCs cases according to PD-L1^High^ expression and indicate that PD-L1 is mostly upregulated in tumors enriched of stem-like features.

### PD-L1 expression is increased in TNBCSCs

**Fig. 2:**
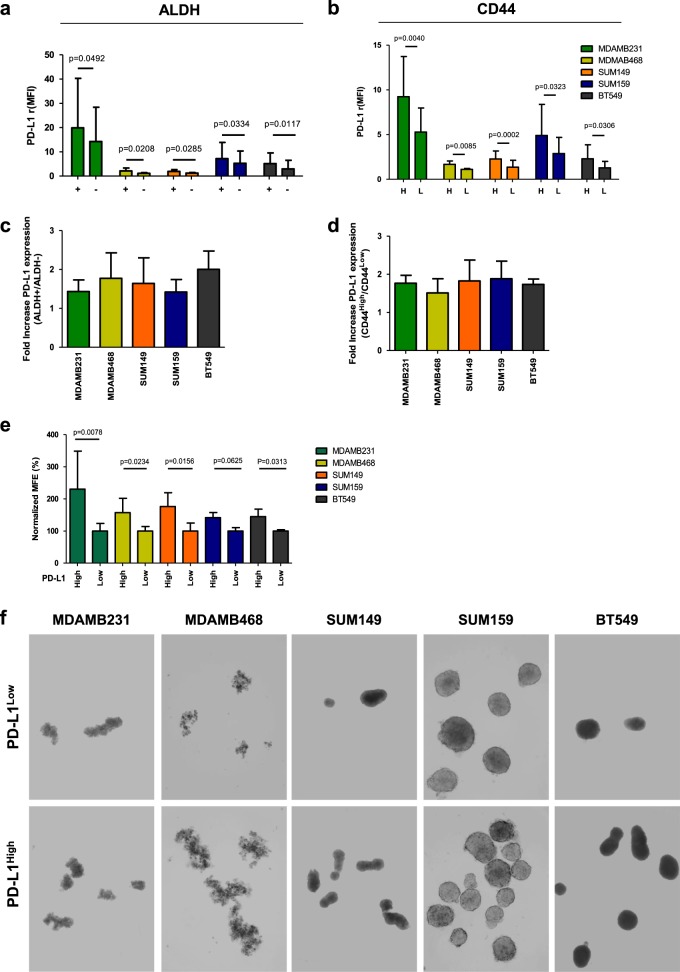
PD-L1 is upregulated in CSC compartments of human TNBC. **a** Evaluation of PD-L1 expression levels by FACS analysis (*Relative median fluorescence intensity* {*rMFI*}) in MDAMB231, MDAMB468, SUM149, SUM159, and BT549 cells gated for ALDH+ and **b** CD44^High^ CSCs biomarkers**. c** Fold increase of PD-L1 protein expression in ALDH+ and **d** CD44^High^ *versus* the ALDH- and CD44^Low^-counterparts. Columns bars, mean ± SD (*n* ≥ 4). Significance was calculated by a two-tailed paired *t*-test. **e** Normalized MFE% of MDAMB231, MDAMB468, SUM149, SUM159, and BT549 cells sorted according to high *versus* low PD-L1 expression. Columns bars, mean ± SD (*n* ≥ 3). Significance was calculated by a two-tailed paired *t*-test. **f** Mammosphere generation in all MDAMB231, MDAMB468, SUM149, SUM159, and BT549 cells sorted according to high *versus* low PD-L1 expression. Spheres formed after 7 days of incubation (magnification ×10)

To evaluate PD-L1 expression in the TNBCSC compartments, we used FACS analysis to examine the PD-L1 expression in the gated epithelial-ALDH-positive (ALDH+) (Fig. 2a) and mesenchymal-CD44-positive (CD44^High^) (Fig. 2b) subsets of MDAMB231, MDAMB468, SUM149, SUM159, and BT549 cell lines *versus* their ALDH-negative (ALDH−) and CD44^Low^ (L) cell counterparts. PD-L1 was found significantly enriched in all tested ALDH+ and CD44^High^ (H) cell compartments (Fig. 2a, b), with an increase in PD-L1 expression ranging from 1.5- to 2.5-fold in both ALDH+ and CD44^High^ *versus* ALDH− and CD44^Low^ counterparts (Fig. 2c, d; Supplementary Fig. S4). Then, using flow cytometry, we sorted the above TNBC cell lines according to PD-L1 expression level (High *versus* Low) (Supplementary Fig. S5) to determine their ability to form mammospheres (MFE%) (Fig. 2e). PD-L1^High^ TNBC cells formed a significantly greater number of mammospheres than PD-L1^Low^ cells (Fig. 2e, f), with the exception of SUM159 cells, which showed only a trend toward significance (*p* = 0.0625) (Fig. 2e, f).

To further sustain the higher expression of PD-L1 in the CSC compartments, we examined the tumor-forming ability of the murine SN25A mammary tumor cells [30] sorted according to PD-L1^High^ *versus* PD-L1^Low^ expression (Table 1) within the gate of SCA-1-positive cells, a murine biomarker for CSCs [31] (Supplementary Fig. S6), and injected them at two different dilutions (10^3^ and 10^2^) into the mammary fat pad of BALB/c mice. We observed that only PD-L1^High^ tumor cells injected at a number of 10^3^ grew in 75% of mice (first tumor by day 35, all tumors within day 65), while PD-L1^Low^ cells did not become established in vivo at the same cell multiplicity. In addition, the extreme dilution assay (ELDA) estimated a higher CSC frequency in PD-L1^High^ (1/873) than in PD-L1-^Low^ (>1469) SN25A cells, providing further evidence for enrichment of the CSC subpopulation in PD-L1-positive cells. Specifically, pairwise tests for differences in stem cell frequencies revealed a significant CSC enrichment in PD-L1^High^ *versus* PD-L1^Low^ (*p* = 0.00737) cell compartment.

**Table 1:** In vivo tumor-forming ability (outgrowths/injections %) of SN25A cells gated for SCA-1 expression

Injected cells (no.)	PD-L1^High^ outgrowths/injections (%)	PD-L1^Low^ outgrowths/injections (%)
10^3^	6/8 [75]	0/4 (0)
10^2^	0/8 (0)	0/4 (0)
ELDA (95% CI)^2^	1/873 (1/386-1/1976)	>1469 (Inf-Inf)

**Fig. 3:**
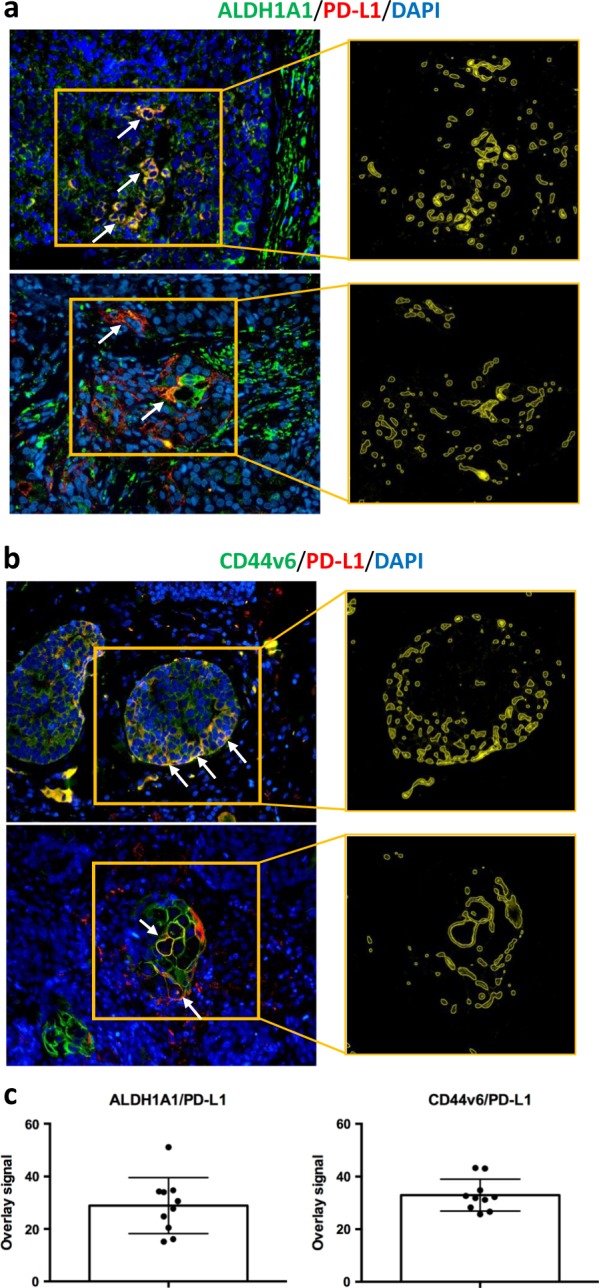
PD-L1 is expressed in ALDH1A1- and CD44v6-positive elements of human TNBC cases. **a** Representative microphotographs of double-marker immunofluorescence for ALDH1A1 (green signal) and PD-L1 (red signal) in TNBC FFPE sections and quantification of overlay signals (yellow; see arrows) corresponding to ALDH1A1 and PD-L1 co-expression. **b** Representative microphotographs of double-marker immunofluorescence for CD44v6 (green signal) and PD-L1 (red signal) in TNBC FFPE sections and quantification of overlay signals (yellow; see arrows) corresponding to CD44v6 and PD-L1 co-expression (See Supplementary Table S2). **c** Bar plots showing the overlay signals between ALDH1A1/PD-L1 (left subpanel) and CD44v6/PD-L1 (right subpanel)

The potential association of PD-L1 expression with intra-tumor human ALDH1A1-positive (Fig. 3a) and CD44v6-positive (Fig. 3b) TNBC cells was evaluated by IHC in 94 invasive ductal TNBCs. The expression of the two markers was scored according to the percentage of positive morphologically atypical elements (Supplementary Table S1). Cases were stained for PD-L1 using double-marker immunofluorescence (Fig. 3a, b). We observed that ALDH1A1-positive or CD44v6-positive atypical cells frequently also expressed PD-L1 (Fig. 3c). In cases in which in situ foci were evident, the fraction of ALDH1A1-/PD-L1-positive malignant cells was preferentially localized in pseudo-basal areas (Fig. 3a). Overall, our analyses unveiled a potential enrichment of PD-L1 expression in the CSC compartment of human TNBCs, supporting PD-L1 expression as a potential marker of stemness in TNBCs.

To further confirm the association between immunophenotypical stemness features and PD-L1 expression, double-marker immunofluorescence analyses were performed by quantifying the amount of marker co-localization through an *ad hoc* software tool (Supplementary Table S2) [32, 33].

### Interaction between WNT signaling and PD-L1 expression

**Fig. 4:**
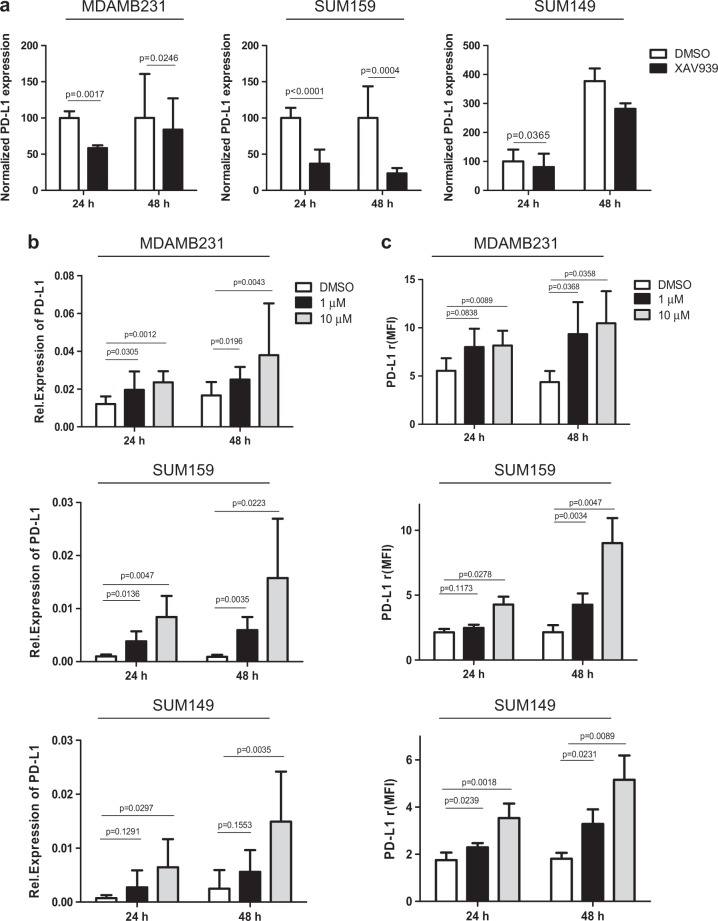
In vitro modulation of PD-L1 expression according to WNT inhibition (XAV939) or activation (CAS 853220-52-7). **a** qRT-PCR analyses of PD-L1 transcript expression evaluated in MDAMB231, SUM159, and SUM149 cells treated with the WNT inhibitor XAV939 (50 µM) or the diluent DMSO for 24 and 48 h. The values were normalized on the PD-L1 expression in control samples. Columns bars, mean ± SD (*n* = 3). Significance was calculated by a two-tailed paired *t*-test. **b** qRT-PCR analyses of PD-L1 transcript expression in MDAMB231, SUM159, and SUM149 cells treated with the selective WNT agonist CAS-853220527 at 1 and 10 µM or the diluent DMSO for 24 and 48 h. Columns bars, mean ± SD (*n* = 3). Significance was calculated by a two-tailed paired *t*-test. **c** FACS analyses of PD-L1 expression in MDAMB231, SUM159, and SUM149 cells treated with the selective WNT agonist CAS-853220527 at 1 and 10 µM or the diluent DMSO for 24 and 48 h. Columns bars, mean ± SD (*n* ≥ 4). Significance was calculated by a two-tailed paired *t*-test

Because the WNT signaling pathway is known to play a crucial role in the regulation of stem cells [34], we investigated whether WNT might be implicated in PD-L1 expression by TNBCSCs. To achieve this goal, we first analyzed the changes in WNT gene expression in PD-L1^High^/SS^High^ TNBCs (*n* = 36). As shown in Supplementary Fig. S7a and b, using an integrative pathway analysis, we observed relevant WNT-related pathways, such as, the downmodulation of upstream negative regulators of WNT (SPFRP4, SPFRP2, EGR1, and WIF1) and an upregulation of a set of downstream WNT effectors (CUL1, RUBL1, TCF/LEF, MYC, MMP7). Then, we analyzed the co-expression patterns of genes annotated in the WNT pathway (GO:0016055) in the Ital-Mex cohort. The molecular data for WNT signaling-related genes, analyzed as a correlation matrix, indicated a significantly higher number of positive or negative co-expression patterns of WNT-related genes in PD-L1^High^ than in PD-L1^Low^ TNBC (Supplementary Fig. S7c), thus suggesting the activation of the WNT signaling pathway in association with PD-L1 expression levels. These findings confirm the robust interactions networks between characterized genes and components of the WNT transcriptional regulatory network in PD-L1^High^ tumors, supporting the implication of a coordinated transcriptional landscape of PD-L1 and WNT gene sets. To determine whether the pharmacological modulation of WNT pathway could affect PD-L1 expression, we treated the MDAMB231, SUM159, and SUM149 cell lines with XAV939, a tankyrase inhibitor that stabilizes AXIN and enhances β-catenin destruction [22]. As shown in Fig. 4a, XAV939 significantly decreased PD-L1 expression in all tested cell lines at 24 and 48 h (albeit to different extents) compared with the internal control DMSO (diluent) without impairing the frequency of ALDH + CSCs (Supplementary Figure S8) or the expression of CD44 (Supplementary Figure S9). Moreover, to sustain the presence of a functional interaction between PD-L1 expression and the WNT signaling pathway, we treated the same cells for 24 and 48 h with 1 and 10 µM CAS 853220-52-7, a WNT agonist that mimics the effects of WNT ligand, and evaluated PD-L1 protein and transcript modulation. In keeping with our findings, we found that the WNT agonist significantly increased PD-L1 expression both at transcript (Fig. 4b) and protein levels (Fig. 4c and Supplementary Fig. S10) in all target cells tested compared with the DMSO control at each time point. Similarly, we did not observe any modulation of the CSC frequency also by treating with CAS 853220-52-7 (Supplementary Figure S11 and S12). To reinforce the data shown in Fig. 4, we treated the cells with a second WNT inhibitor (LGK-974) and, in parallel, with an additional WNT agonist (SKL2001), still confirming the capability of these compounds to decrease or increase PD-L1 expression, respectively (Supplementary Figure S13 and S14). To further validate these observations, we took advantage of a public expression dataset (GSE40715) to evaluate the stem-like Lin-CD29highCD24 + and differentiated/bulk Lin-CD29 + CD24+ cell compartments of the murine TNBC *Apc*1572T/+ cell model driven by WNT activity. Similar to our previous observations, the subpopulation highly enriched for mouse mammary stem cells and with a more active WNT signaling was found to overexpress PD-L1 (Supplementary Fig. S15).

### PD-L1-positive TNBCSCs interact with infiltrating immune cells

**Fig. 5:**
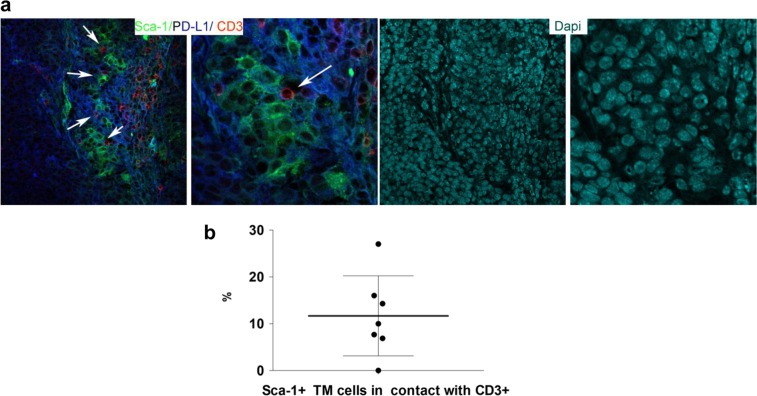
In vivo interaction between murine mammary CSCs with immune cells. **a** Representative confocal microscopy analysis of SN25A cells grown in the parental BALB/c mice after staining for PD-L1 (blue), CD3 (red), and Sca-1(green) shows putative PD-L1 + Sca-1 + CSC in close contact with CD3+ cells (see arrows) (×20, left panels; ×40, right panels). **b** Dot plot representing the frequency (%) of CSC contacting CD3+ cells calculated as fraction between the number of Sca1 + PD-L1 +  tumor (TM) cells in contact with CD3 and the total number of Sca1 + PD-L1+ cells in the sample

**Fig. 6:**
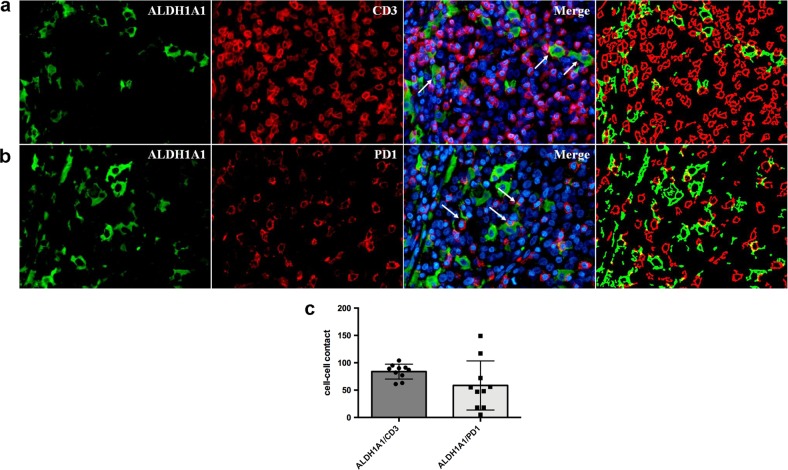
Interaction between human CSCs with immune cells in TNBC cases. **a** Immunofluorescence analysis showing the direct spatial interaction (see arrows) between ALDH1A1+ cells (green) and CD3+ (red) in TNBC FFPE sections. **b** Immunofluorescence analysis showing the direct spatial interaction between ALDH1A1+ and PD1 + T cells (red) in TNBC FFPE sections. **c** Dot plots showing the quantification obtained through a software-mediated segmentation of the different cell populations stained for ALDH1A1 (green) and CD3 (red), or PD1 (red) signals (See Supplementary Table S3)

To evaluate the potential roles exerted by PD-L1 upregulation in protecting TNBCSCs from immune T cell attack, we first analyzed the occurrence of an in situ direct interaction between PD-L1^High^ CSCs and T cells. We performed a confocal microscopy analysis for the tumor-restricted expression of PD-L1 and SCA-1, and for the T-cell marker CD3 on murine mammary tumors grown following the orthotopic injection of SN25A cells into immunocompetent syngeneic BALB/c mice. As shown in Fig. 5a and b, the close contact between CD3-positive cells and PD-L1-positive/SCA-1-positive tumor elements strongly supports our hypothesis. In keeping with these results, we also identified a close contact between ALDH1A1-positive malignant cells and CD3- (Fig. 6a, b) or PD1-expressing (Fig. 6a, b) T cells in human TNBCs in situ, which further indicates the capability of the CSCs to directly interact with T cells and thus potentially influence the immune response to cancer. This interaction was also quantitatively evaluated by an *ad hoc* software tool, which calculated the number of CD3 or PD1-expressing elements in direct contact with ALDH1A1-positive cells, in ten high power magnification fluorescence images (Supplementary Table S3 and Fig. 6b).

## Discussion

BC development and progression is dependent on a complex system of different cooperating factors, including genetic and epigenetic alterations, the existence of a cell subpopulation that can influence the tumor therapeutic response and contribute to chemo- and radio-therapy resistance and tumor relapse (CSCs), as well as components from the tumor microenvironment, such as immune and stromal cells [18, 35]. An increasing number of molecular signaling “hubs” that include the JAK/STAT, Hedgehog, WNT, Notch, NF-κB, PI3K, and PTEN pathways have been discovered to become dysfunctional in CSCs, contributing to their maintenance, and are now entering clinical trials [36].

The role of the immune system in the eradication of cancer is well established in light of the major success in the blockade of the T cell inhibitory molecule, PD-L1, which is expressed by cancer cells to drive an immunosuppressive microenvironment [7, 8, 37]. Although checkpoint inhibitor therapies have been a breakthrough in the treatment of several cancer types, only a small subset of TNBC patients derives substantial benefit and has shown a durable clinical benefit from these therapies [8]. Since increasing evidence is revealing that the overexpression of tumor-associated PD-L1 is a weak and insufficient predictive biomarker of therapy response in different cancer populations, the identification of solid predictors is an urgent and challenging clinical need, to optimize treatment selection [16].

Thus, a relevant oncological goal is the design of new cancer immunotherapy strategies able to increase the immunogenicity of cancer cells with the aim to extend the durable benefits of disease progression and recurrence prevention.

In this particular scenario, our study characterized in detail the biological interplay of two important components of the CSC programs and tumor immunity in TNBCs as the WNT signaling pathway and PD-L1 expression in the stem-like subpopulation. First, the transcriptional landscape characterization of TNBCs cohort stratified by PD-L1 expression levels revealed a significant enrichment of many immune-related and stem-like WNT signaling gene pathways in the PD-L1^High^ cases. Gene sets associated with tumor immunity and stem cell signaling/maintenance suggest that PD-L1 expression patterns may play a role in not only the immunosuppressive status but also in the stemness status. In particular, we observed a significant positive correlation between the expression of SS features and PD-L1 expression only in the SS^High^ PD-L1^High^ TNBC subset. Moreover, the increase in PD-L1 expression was not always correlated to enriched stemness features, as we identified a SS^Low^ PD-L1^High^ subgroup. In parallel, the expression of claudin-low signature genes, a phenotype known to contribute to the activation and progression of the EMT program, was strongly upregulated in tumors with high PD-L1 expression. Thus, PD-L1 expression has been observed in different tumor subpopulations in TNBCs, possibly driven by different biological signaling pathways according to each cell compartment. Because the relationship between stemness-like cells and the PD-L1 transcriptional landscape is currently unclear, we wished to determine the significance of the synchronicity of these biological characteristics and their tumor driver pathways.

In accordance with the data previously reported in BC cells by Alsuliman et al. [38], who demonstrated that PD-L1 expression is induced upon the EMT process, and by Almozyan, who revealed that PD-L1 is able to sustain stemness in BC [19], our results provide evidence that PD-L1 is mostly upregulated not only in TNBCs enriched for EMT features but also in a stem-like phenotype. Additionally, the higher and significant expression of PD-L1 in ALDH+ and CD44^High^ CSC cell compartments *versus* ALDH- and CD44^Low^ counterparts in the five TNBC cell lines, as well as the significantly greater capacity of their sorted PD-L1^High^ *versus* PD-L1^Low^ subsets to generate mammospheres, consistently sustained our molecular analyses. To corroborate these findings, we tested, in parallel, the in vivo tumor-forming ability of SN25A cells sorted for PD-L1^High^ or PD-L1^Low^ expression and injected them into mice at the numbers of 10^3^ or 10^2^. Consistent with the in vitro data, we detected in vivo tumor uptake only by injecting mice with 10^3^ PD-L1^High^ cells. These pre-clinical data were further supported by the analysis of double-marker immunofluorescence in human TNBC cases that allowed us to reveal the intra-tumor coexistence of neoplastic elements co-expressing both ALDH1A1 with PD-L1 and CD44v6 with PD-L1 biomarkers. Thus, also in human samples, we showed a potential enrichment of PD-L1 in both epithelial (ALDH1A1-positive) and mesenchymal (CD44v6-positive) CSC subsets. Therefore, the expression of PD-L1 by stem-like compartments can partly explain the resistance of CSCs to anti-tumor immune attack, as also speculated by others in head and neck and colorectal cancer models [9, 21]. In this context, the short-term duration and reduced response in TNBC patients treated with immune checkpoint inhibitors (ICB) could be partly due to the upregulation of different immune-escape biological mechanisms in the CSC subset and to the heterogeneous expression of PD-L1 in TNBC differentiated/bulk tumor cells, which could, in turn, function as “decoys” for ICB activity by decreasing their efficacy over the less frequent CSC compartment.

Thus, to handle these issues and to define mechanism(s) that modulate PD-L1 expression on TNBCSCs, we focused our attention on the regulatory signaling pathways of both CSC activities and PD-L1 expression on CSCs to target the CSC population and render them less prone to mediating immunotherapy resistance.

As stated, a number of developmental pathways are critical for the maintenance of CSC activities [36]. In this context, WNT signaling regulates a variety of cellular processes, including cell fate, differentiation, proliferation and stem cell pluripotency, and its aberrant signaling is a hallmark of many cancers [34]. Specifically, a dysfunctional canonical and non-canonical WNT signaling is characteristic of TNBCs, either in the establishment of TNBC tumorigenesis or metastasis [39–41]. In particular, it has been reported that an enrichment of canonical Wnt/beta-catenin signaling pathway is associated with a poor clinical outcome of TNBC patients [42, 43], and the non-canonical signaling pathway has been implicated in CSC maintenance [43]. In light of these studies and the evidence that silencing WNT reduces the MFE of breast CSCs in vitro [44], and taking into consideration the relevant need to increase cancer cell immunogenicity, we herein evaluated the potential to “knock-down” PD-L1 expression on CSCs by modulating the activity of its regulatory signaling pathway. To achieve this goal, our research provided evidence that WNT signaling dysfunction impacted tumor immunomodulatory properties, as its activation or inhibition by specific agonists or inhibitors, respectively, significantly altered PD-L1 expression levels. Similarly, our in silico analysis of a public murine dataset (GSE40715) allowed us to observe that the CSC compartment enriched in WNT activation was coupled to the upregulation of PD-L1. In this context, it has been also reported that post-translational modification of PD-L1 could regulate cancer cell-mediated immunosuppression [45, 46]. Specifically, very recently, Li et al. demonstrated that glycosylation of PD-L1, a phenomenon they found mainly associated to the PD-L1-positive tumor compartment, is a crucial event for PD-L1/PD1 interaction and immunosuppression in TNBC models [47]. In addition, Hsu et al. revealed an upregulation of PD-L1 glycosylation in breast CSCs due to the activation of beta-catenin/STT3 axis, which is mediated by EMT biological process. These data are consistent with our results and establish the WNT-signaling pathway as the main mechanism upregulating PD-L1 expression in the TNBCSC compartment [48].

In addition, we also found a direct interaction in situ between PD-L1^High^ SCA-positive CSC elements and CD3-positive T cells in murine tumor samples. These findings were reproducible in human TNBC cases, in which we visualized a direct contact of ALDH1A1-positive tumor elements with CD3- and PD1-positive T cells. Interestingly, we observed that SS^High^ and PD-L1^High^ TNBCs were in close contact with CD3-positive T cells, suggesting the presence of ineffective anti-tumor immunity. Overall, this study showed that the activation of WNT signaling pathway induced PD-L1 expression in the CSC compartment and that its expression, in turn, participated in the stem-like TNBC phenotype. Therefore, WNT inhibition inducing a “silencing” of PD-L1 expression in the CSC component should favor the functional reversion of inactivated CD3 + T cells.

In summary, our studies directed toward understanding a role for CSCs in immunotherapy resistance of TNBC support the candidacy of WNT signaling in governing and sustaining PD-L1^High^ expressing CSCs and its potential targeting to trigger an effective anti-tumor immune response.

## Materials and methods

### Patients and gene expression profile

Our patient cohort was collected at the Fondazione IRCCS Istituto Nazionale dei Tumori of Milan (INT) and Fundación Mexicana de Fomento para la Prevención Oportuna del Cáncer de Mama, A.C., (FUCAM) after approval from the ethics committees (*n* = 158). All patients provided written consent for the use of their biological materials for future investigations. The clinical inclusion criteria for the FFPE tumor sections were as follows: patients who were treatment naïve; at least 60% tumor cell content; and triple-negative status for the immunochemistry markers estrogen receptor, progesterone receptors and HER2. Global gene expression was assessed by the Human Transcriptome Array 2.0 platform (Affymetrix, Central Expressway, Santa Clara, CA, USA). The hybridization, washing and scan procedures were performed according to the protocol proposed by the manufacturer. RMA background correction and quantile normalization were performed using the Transcriptome Analysis Console Software (Affymetrix). Gene expression data are accessible at the GEO repository under accession numbers GSE86946 and GSE86945.

### TNBC grouping and characterization

All statistical analyses and data presentations were performed in R software and the bioconductor environment. For enrichment pathway analysis, gene ontology (Biological process annotation) and KEGG and Reactome terms were analyzed in DAVID (https://david.ncifcrf.gov/summary.jsp) and Innate DB (http://www.innatedb.ca/redirect.do?go=batchPw#textbox) data bases over a pre-ranked list of differentially expressed genes in tumors over-expressing PD-L1. To divide the cohorts evaluated according to PD-L1 expression, we defined a cutoff according to the median distribution value: those samples with PD-L1 expression over the median value were considered up-modulated, while samples with a PD-L1 expression value below the median were considered down-modulated. GSEA was performed over the profiled tumors divided by PD-L1^High^ or PD-L1^Low^ levels using the curated gene sets: gene ontology (Biological process) and pathway (KEGG, Reactome, Biocarta) using Java-based software. To define significant differences between biological conditions, a *t*-test using the R software packages was calculated, and a confidence value of 95% (*p* value ≤ 5%) was considered. For grouping, our cohort according to SS and PD-L1 expression, we used the following criteria: (1) SS^High^/PD-L1^Low^, SS higher than the median and PD-L1 expression lower than the median; (2) SS^High^ /PD-L1^High^, SS and PD-L1 expression higher than the median; (3) SS^Low^ /PD-L1^Low^ low, SS and PD-L1 expression lower than the median; and (4) SS^Low^/PD-L1^High^, SS lower than the median and PD-L1 expression higher than the median.

To test the enrichment of the well-described BC phenotype claudin-low, we defined the claudin-score as the continuous mean of the identified up-modulated genes in claudin-low tumors compared with other tumor subtypes published on [27].

### Immunohistochemistry and immunofluorescence

Sections from FFPE TNBC samples were deparaffinized, rehydrated and unmasked using Novocastra Epitope Retrieval Solution, pH 6, 8, and 9 (Novocastra Laboratories, Newcastle upon Tyne, UK) in a PT Link Dako pre-treatment module at 98 °C for 30 min (Dako, Santa Clara, CA, USA). Subsequently, the sections were brought to room temperature and washed with PBS. The sections were incubated with endogenous peroxidase (3% H_2_O_2_) and a specific protein block (Novocastra Laboratories). IHC was performed using the Novolink Polymer Detection System with the mouse anti-human CD44v6 (clone VFF-18, 1:500, pH 9) (Abcam, Cambridge, UK), ALDH1A1 (1:500, pH 6) (GeneTex, Irvine, CA, USA), and rabbit anti-human PD-L1 (clone E1L3N, 1:200, pH 9) (Cell Signaling Technologies) antibodies. The 3-amino-9-ethyl-carbazole (Dako) and 3-3′ diaminobenzidine (Novocastra) was used as chromogenic substrates. The expression of ALDH1A1, CD44v6, and PD-L1 was scored according to the percentage of intensely-positive cells with atypical morphology. Double-marker immunofluorescence analyses were performed in cases in which the fraction of ALDH1A1 or CD44v6 was equal to or lower than 5%, being suggestive of foci of cells with cancer stem-like phenotype.

For double-marker immunofluorescence staining, the tissue samples were incubated with the following primary antibodies: ALDH1A1; CD44v6; PD-L1 (clone 22C3 Dako, 1:50, pH 9 and clone 28-8 Abcam, 1:500, pH 8); CD3 (clone LN10, 1:500, pH 9) (Novocastra Laboratories); and PD1 (clone NAT105, 1:50, pH 8) (Abcam). The following secondary antibodies were used: Alexa Fluor 568-conjugated goat anti-mouse and Alexa Fluor 568-conjugated goat anti-rabbit (1:300) (Life Technologies, Carlsbad, CA, USA); and Alexa Fluor 488-conjugated goat anti-mouse and Alexa Fluor 488-conjugated goat anti-rabbit (1:250) (Life Technologies). Nuclei were counterstained with DAPI (4′,6-diamidin-2-fenilindolo). Slides were analyzed under a Zeiss Axioscope A1, and microphotographs were collected using a Zeiss Axiocam 503 Color with Zen 2.0 software (Zeiss, Oberkochen, DE).

Quantitative analyses of ALDH1A1 and CD44v6 expression in tumor foci was performed using the Positive Pixel Count v9 Leica Software Image Analysis in five non-overlapping fields at low-power magnification (×10). The Supplementary Table S1 shows the high degree of inter-case variation in the percentage of cells expressing CD44v6 and ALDH1A1 indicating that the fraction of cells co-expressing ALDH1A1/PD-L1 or CD44v6/PD-L1 (Supplementary Table S2) is less variable then that the single marker expressing cells (Supplementary Table S1). Significant although variable number of CD3- and PD1-expressing T cells contacts the ALDH1A1 expressing cells as reported in Supplementary Table S3.

### In vivo tumorigenicity

Animal care and experimental procedures were approved by the Ethics Committee for Animal Experimentation of the Fondazione IRCCS Istituto Nazionale dei Tumori and performed in accordance with Italian law (project number INT16/2016). Eight-week-old-female Balb/c mice (*n* = 4 or 8 per group), purchased from Charles River Laboratories (Wilmington, MA, USA), were injected into the mammary fat pad (mfp) with serial dilutions (10^2^ and 10^3^ cells) of SCA-1-positive isolated from (FACS-sorting) SN25A cells. The mice were monitored twice weekly for up to 3 months. The frequency of stem cells between groups and the adequacy of the single hit model were determined using the ELDA web tool for limiting dilution analysis [49].

### Cell treatments with WNT inhibitors/agonists

Single cell suspensions of MDAMB231, SUM159, and SUM149 cells were plated and grown in a 24-well plate at a density of 50,000–70,000 cells per well for 2 days at 37 °C and 5% CO_2_. To evaluate the effects exerted by selective WNT inhibition or activation on tumor-restricted PD-L1 expression both at mRNA and protein levels, cells were treated with the WNT inhibitors XAV939 (Selleckchem, Karl-Schmid-Str.14, Munich, DE) as a single agent at the final concentration of 50 μM for 24 and 48 h, and LGK-974 (Selleckchem) as a single agent at the final concentration of 10 μM for 1 and 3 h, and with 0.1% of the diluent DMSO (Sigma-Aldrich) as a control at 37 °C and 5% CO_2_. In parallel, the same TNBC cells were treated with the specific WNT agonists CAS 853220-52-7 (Santa Cruz Biotechnology, Heildeberg, DE) as a single agent at both 1 and 10 µM, and SLK2001 (Merck, Darmstadt, Germany) as a single agent at both 20 and 40 µM, and with 0.1% of the diluent DMSO (Sigma-Aldrich) as a control for 24 and 48 h.

### Data/statistical analyses

Statistical analyses were performed with GraphPad Prism 5.02 software using an unpaired or paired two-tailed Student’s *t*-test. When *p* < 0.05, the difference between the compared groups was considered significant. The data are presented as the mean ± SD (*n* ≥ 3 technical replicates). The sample size of each distinct experiment is reported in the corresponding figure legend.

## Supplementary information

Supplementary Figure S1

Supplementary Figure S2

Supplementary Figure S3

Supplementary Figure S4

Supplementary Figure S5

Supplementary Figure S6

Supplementary Figure S7

Supplementary Figure S8

Supplementary Figure S9

Supplementary Figure S10

Supplementary Figure S11

Supplementary Figure S12

Supplementary Figure S13

Supplementary Figure S14

Supplementary Figure S15

Amended Supplementary Figure Legends not Highlighted

Supplementary Table S1

Supplementary Table S2_amended

Supplementary Table S3
