# Supplementary figures and images for: WNT signaling modulates PD-L1 expression in the stem cell compartment of triple-negative breast cancer
Source: Oncogene. 2019 Jan 31;38(21):4047–60. doi: 10.1038/s41388-019-0700-2 (PMC6755989; doi:10.1038/s41388-019-0700-2)

**Supplementary Figure S1**

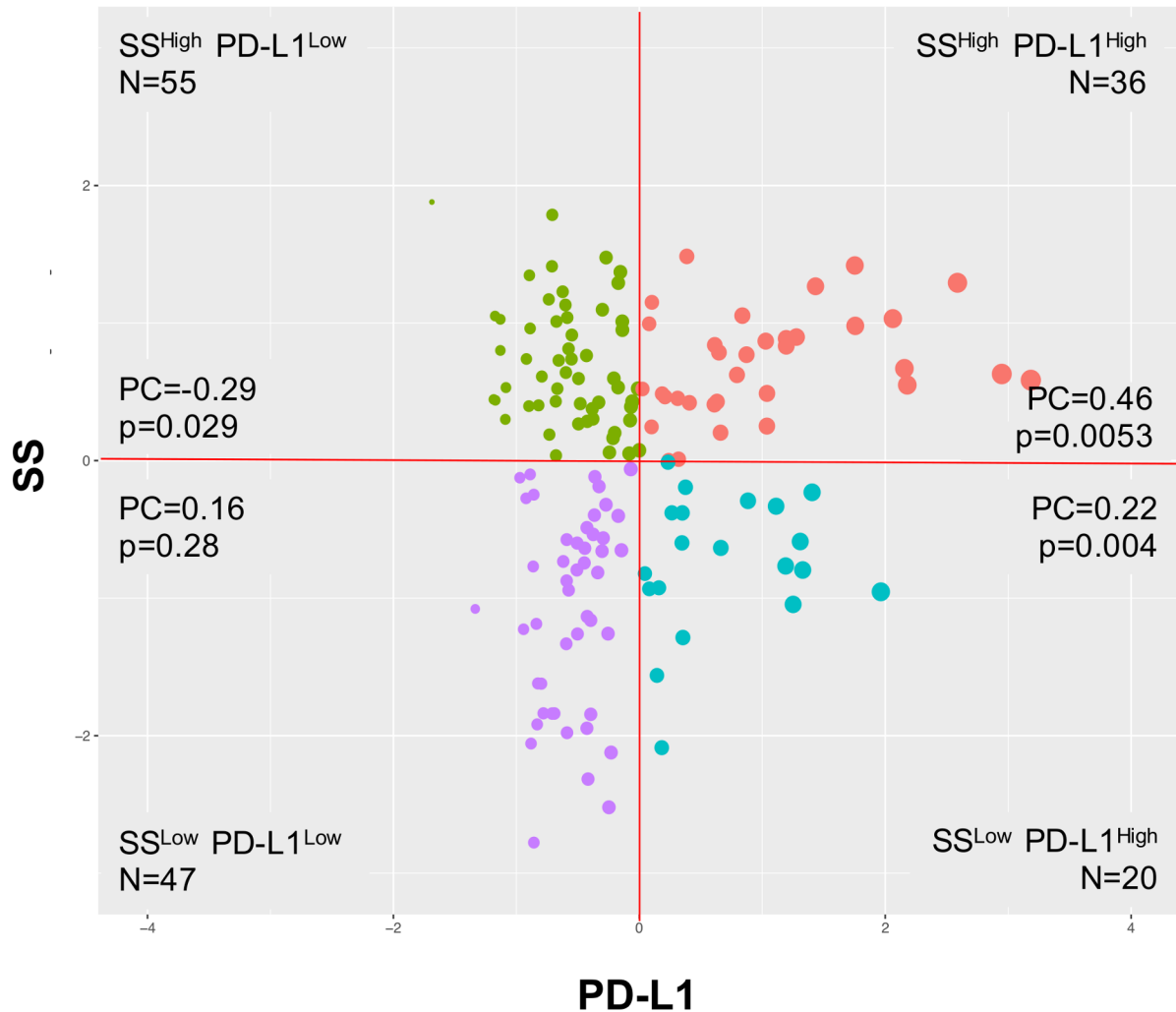

Supplement: Supplementary file 1 — Supplementary Figure S1 [file 41388_2019_700_MOESM1_ESM.pdf]

# Supplementary Figure S2

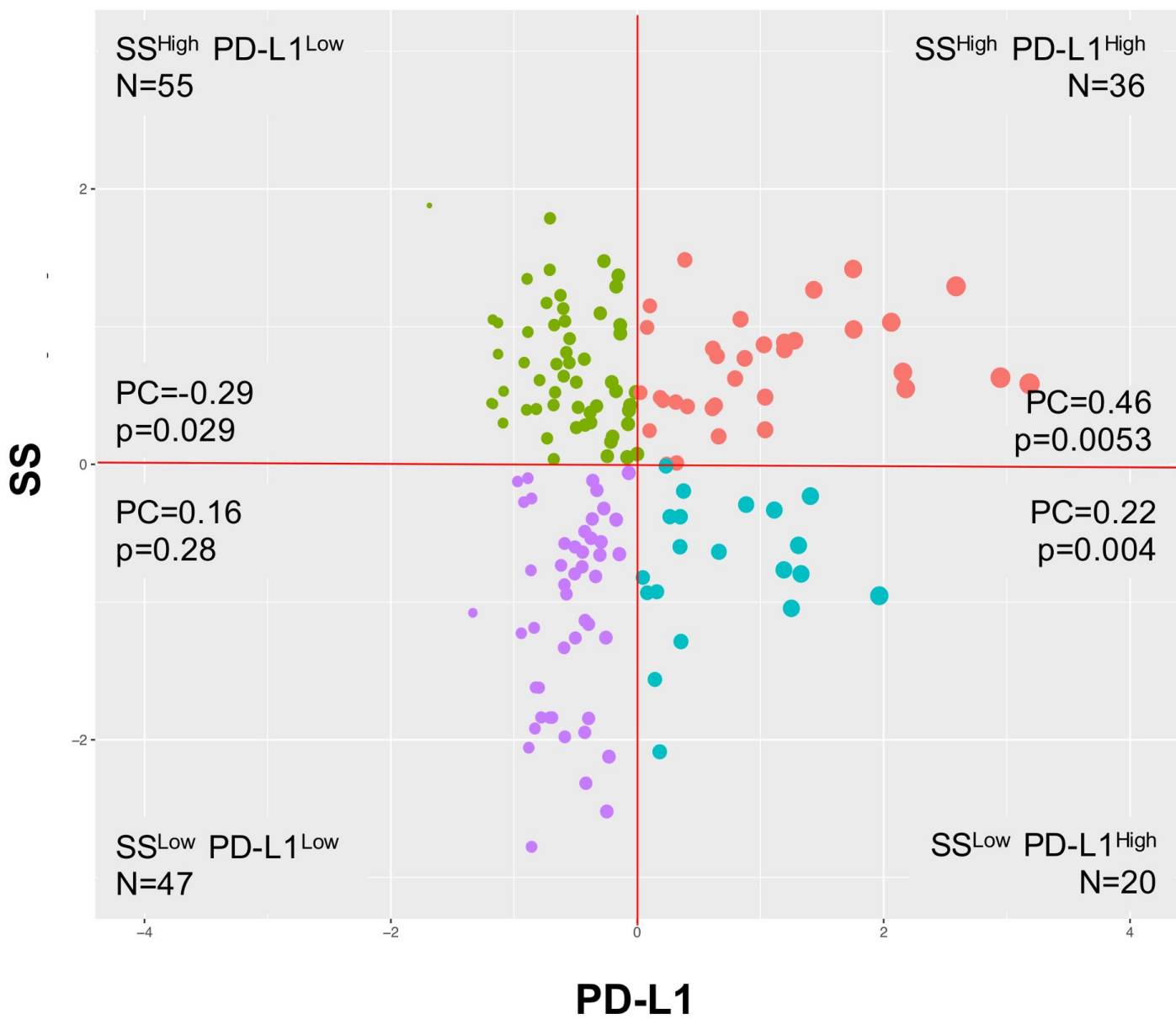

Supplement: Supplementary file 2 — Supplementary Figure S2 [file 41388_2019_700_MOESM2_ESM.pdf]

Supplementary Figure S3

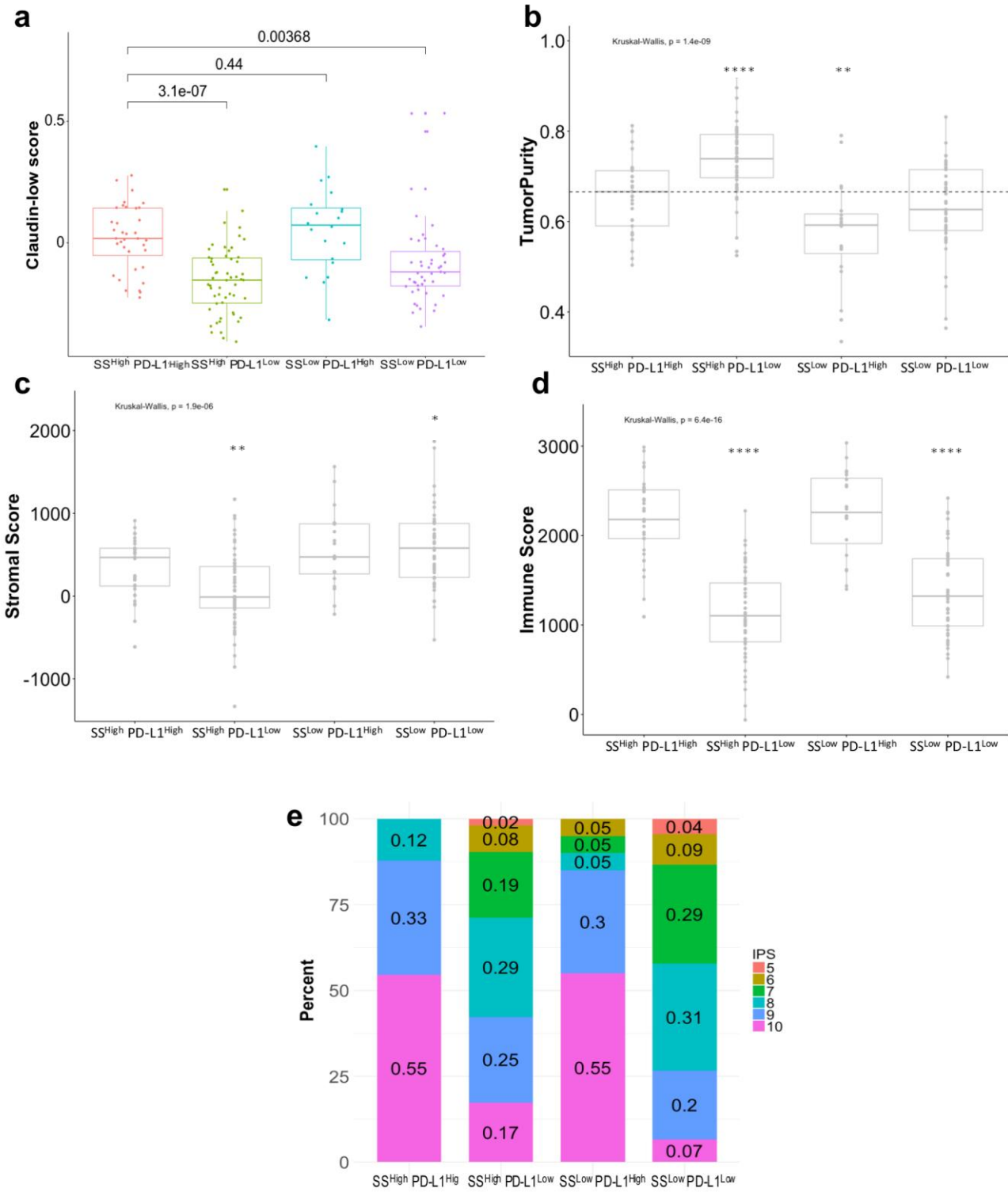

Supplement: Supplementary file 3 — Supplementary Figure S3 [file 41388_2019_700_MOESM3_ESM.pdf]

# Supplementary Figure S4

**a**

## DEAD CONTROL

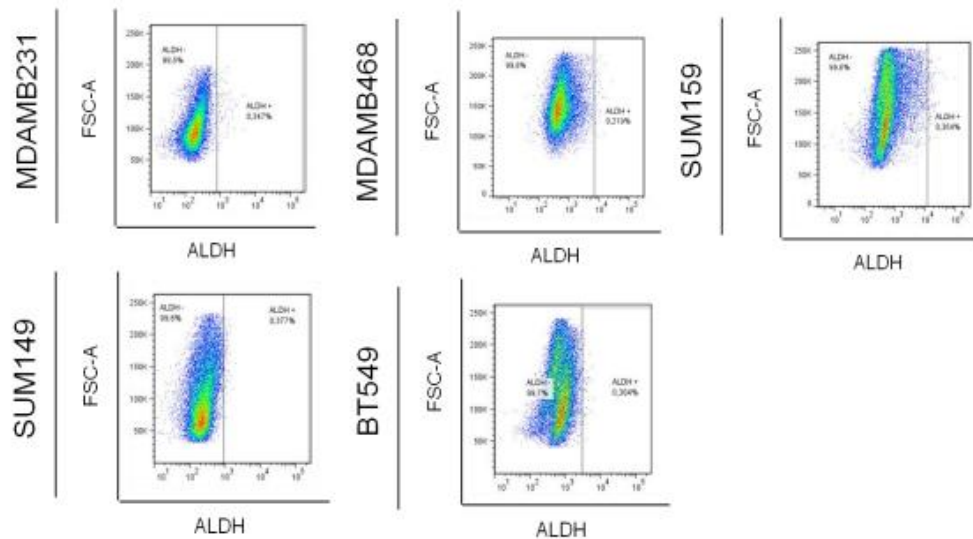

**b**

## ALDH

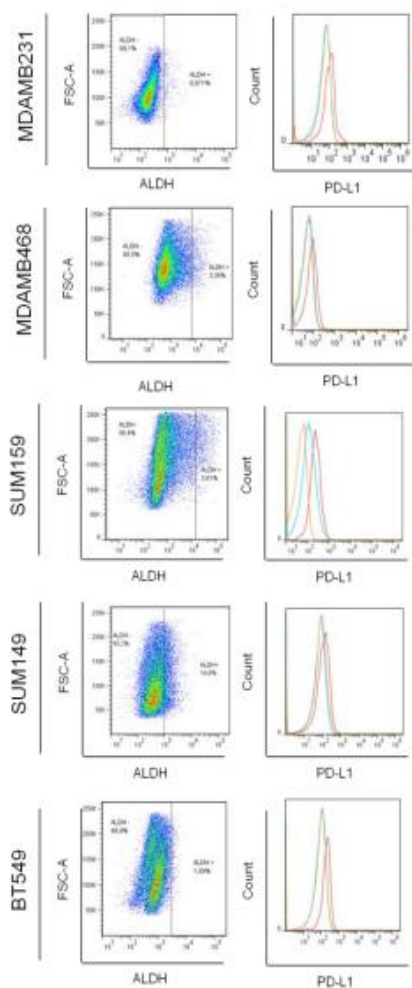

**c**

## CD44

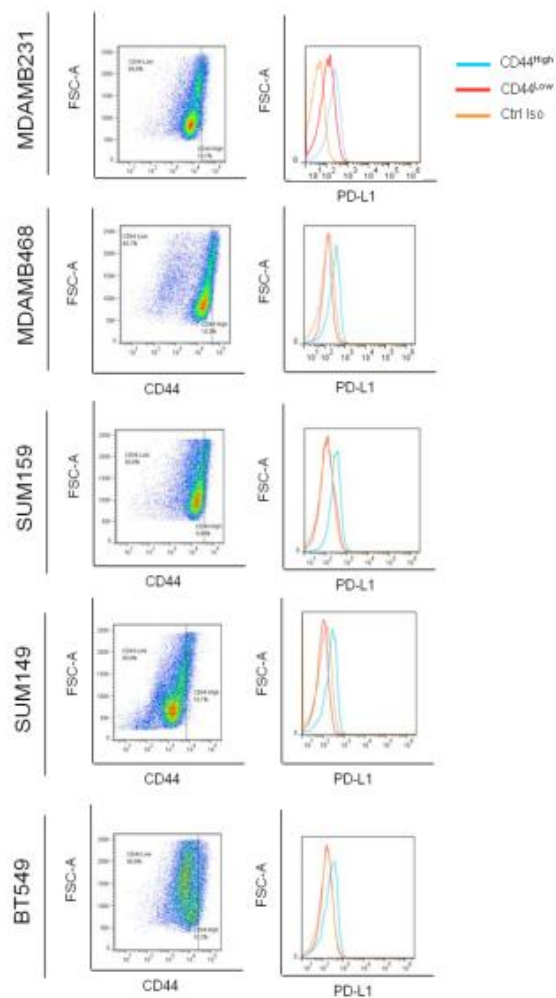

Supplement: Supplementary file 4 — Supplementary Figure S4 [file 41388_2019_700_MOESM4_ESM.pdf]

# Supplementary Figure S5

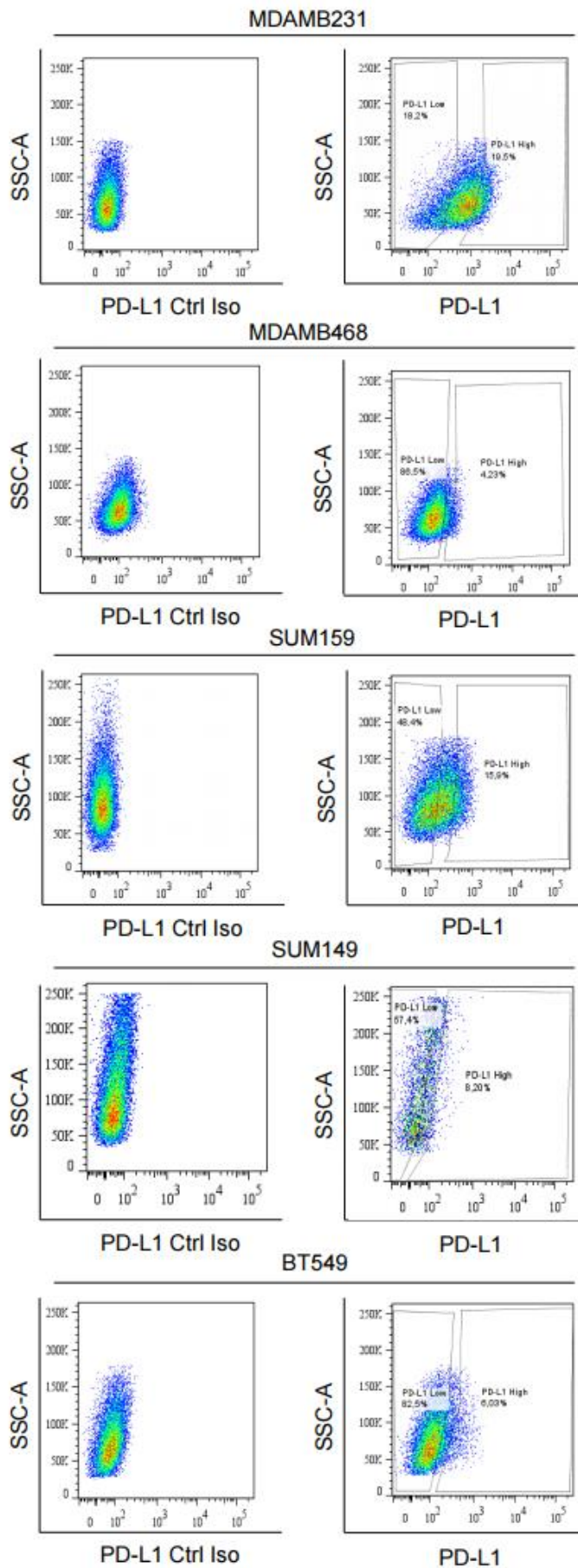

Supplement: Supplementary file 5 — Supplementary Figure S5 [file 41388_2019_700_MOESM5_ESM.pdf]

# Supplementary Figure S6

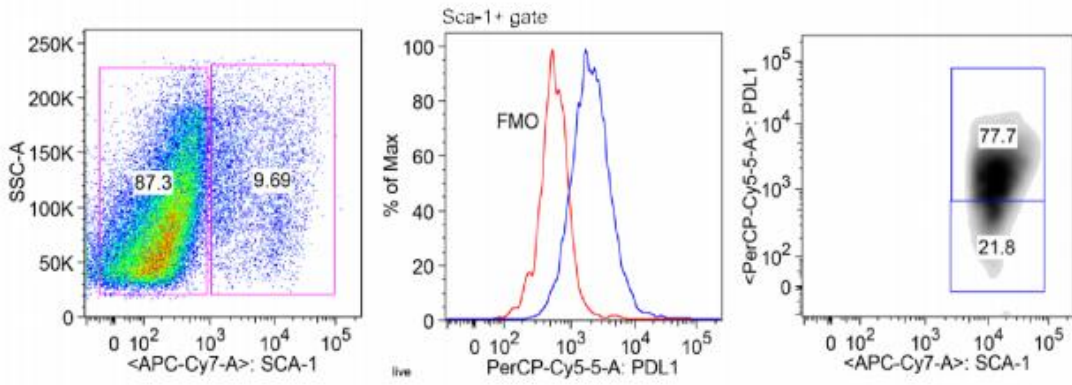

Supplement: Supplementary file 6 — Supplementary Figure S6 [file 41388_2019_700_MOESM6_ESM.pdf]

# Supplementary Figure S7

**a**

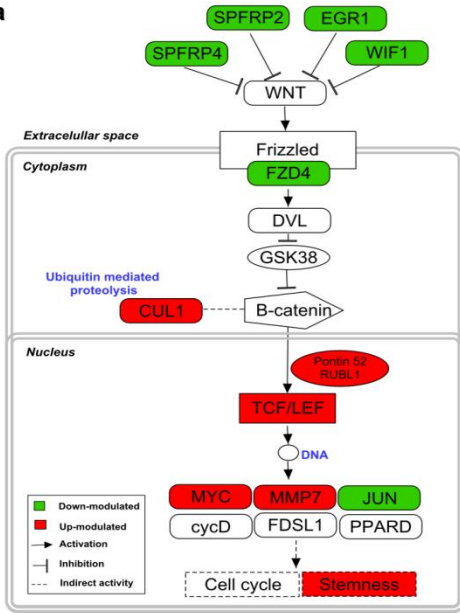

**b**

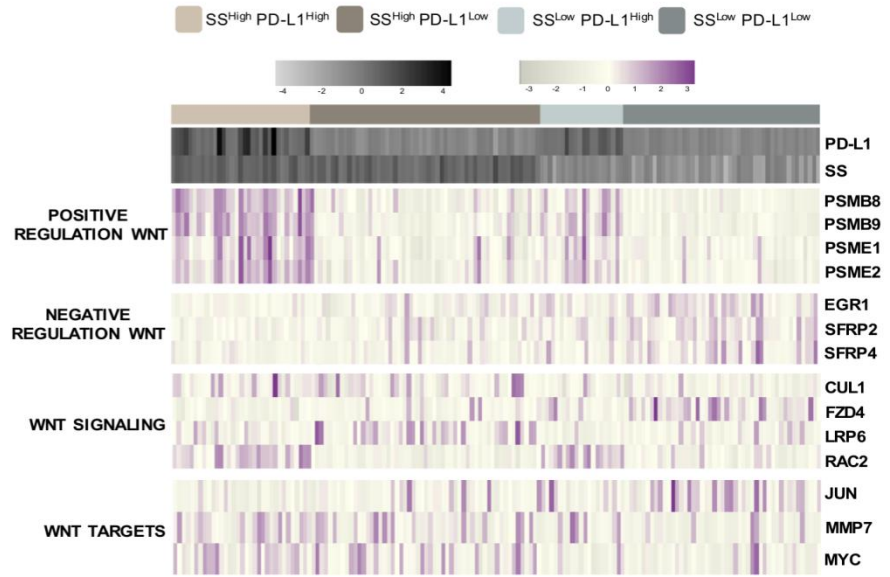

**c**

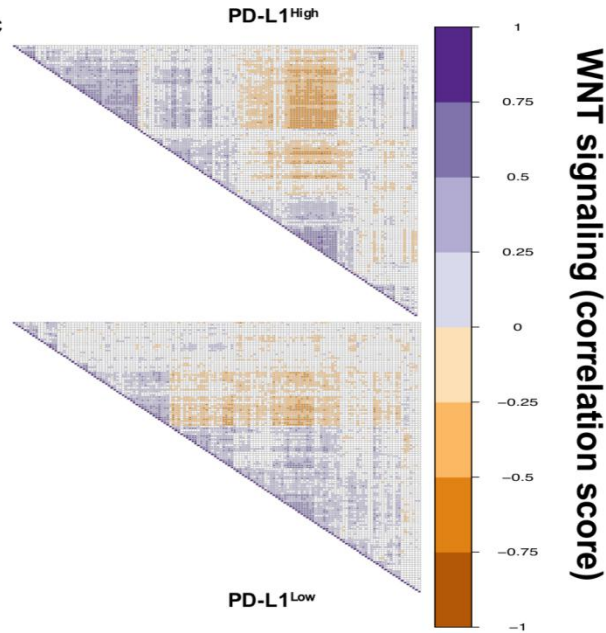

Supplement: Supplementary file 7 — Supplementary Figure S7 [file 41388_2019_700_MOESM7_ESM.pdf]

MDAMB231

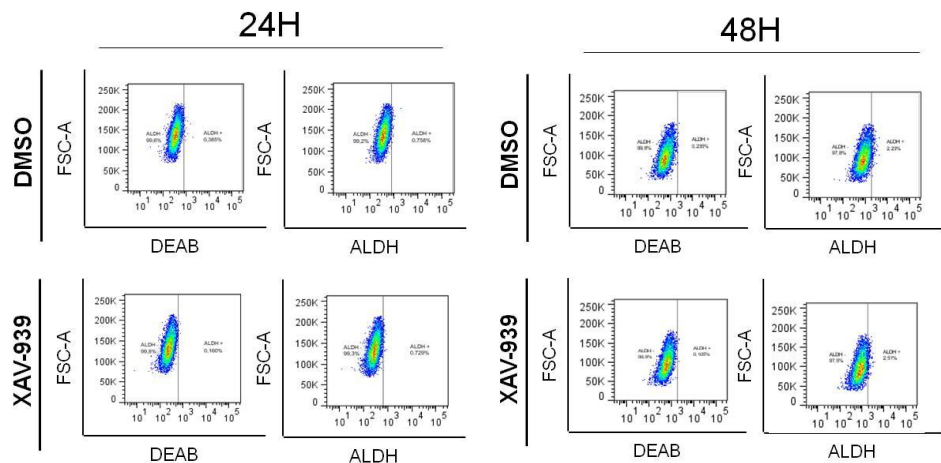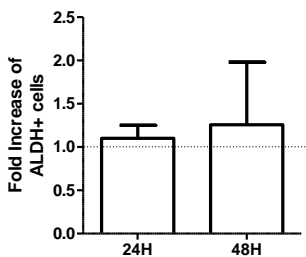

SUM149

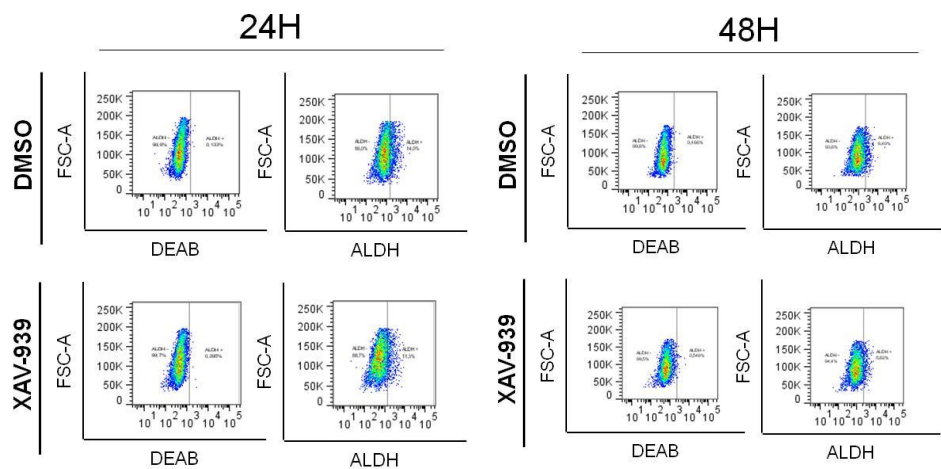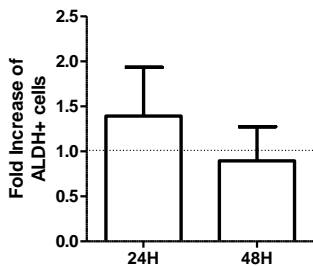

SUM159

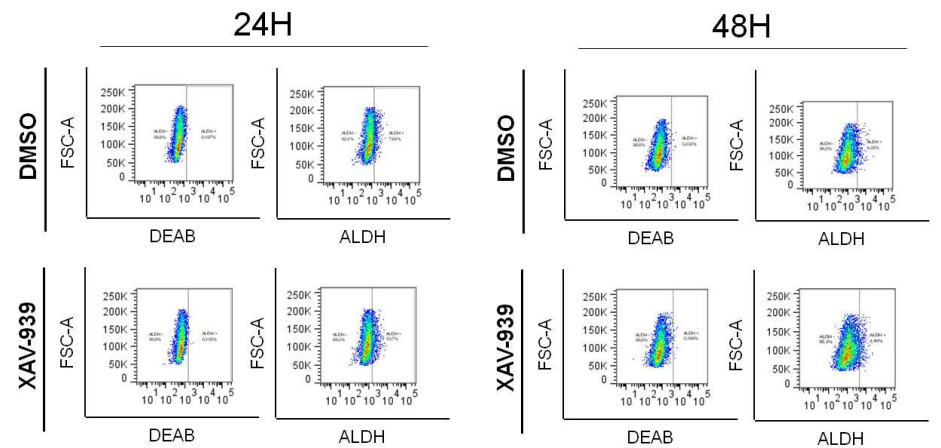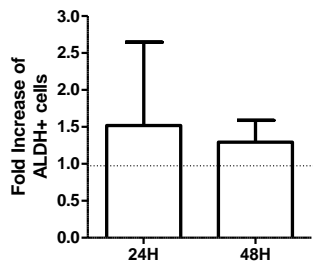

Supplement: Supplementary file 8 — Supplementary Figure S8 [file 41388_2019_700_MOESM8_ESM.pdf]

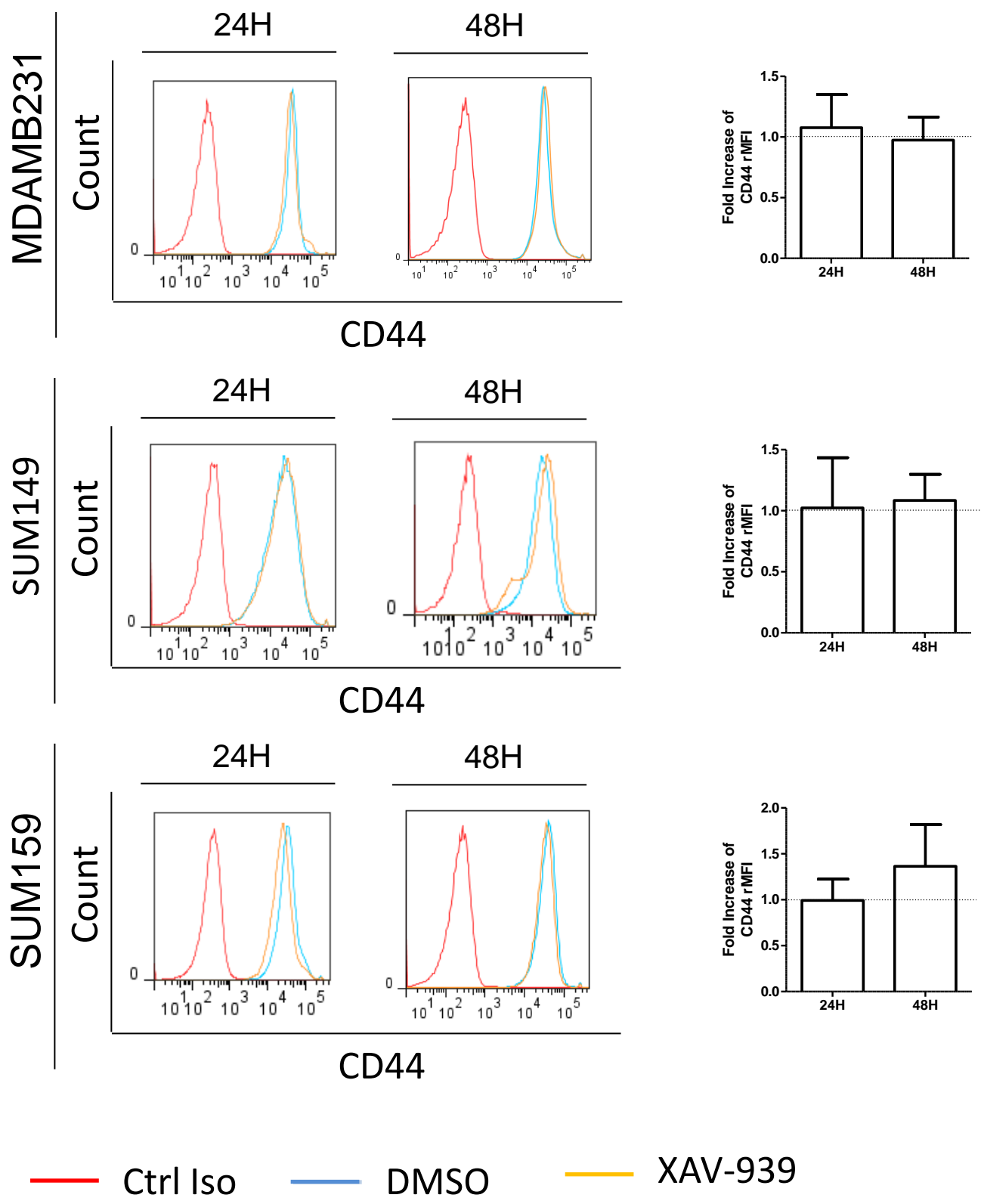

Supplement: Supplementary file 9 — Supplementary Figure S9 [file 41388_2019_700_MOESM9_ESM.pdf]

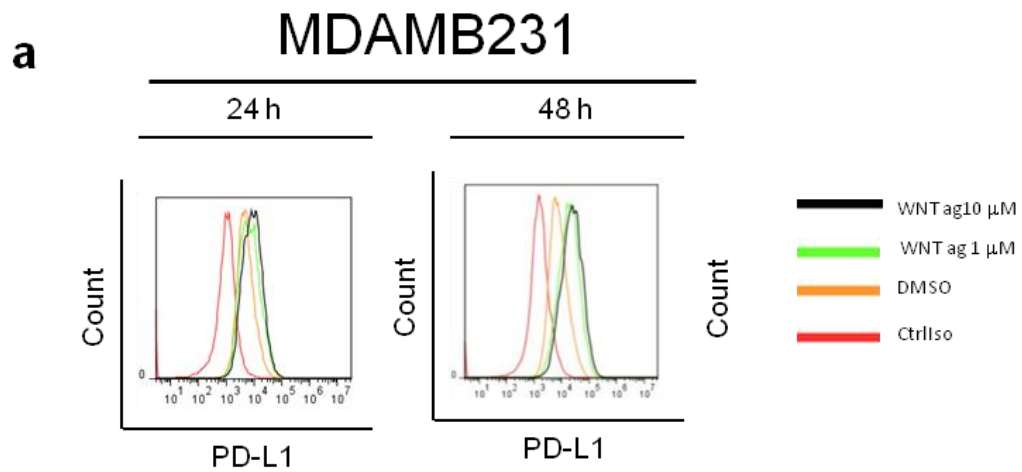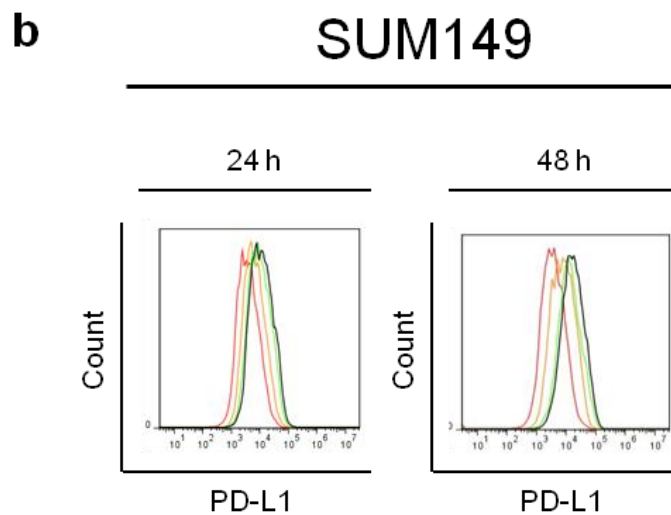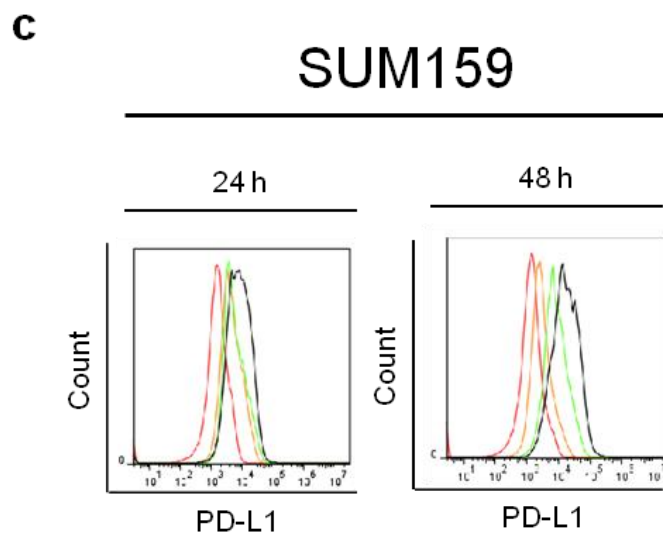

Supplement: Supplementary file 10 — Supplementary Figure S10 [file 41388_2019_700_MOESM10_ESM.pdf]

Supplementary Figure S11

MDAMB231

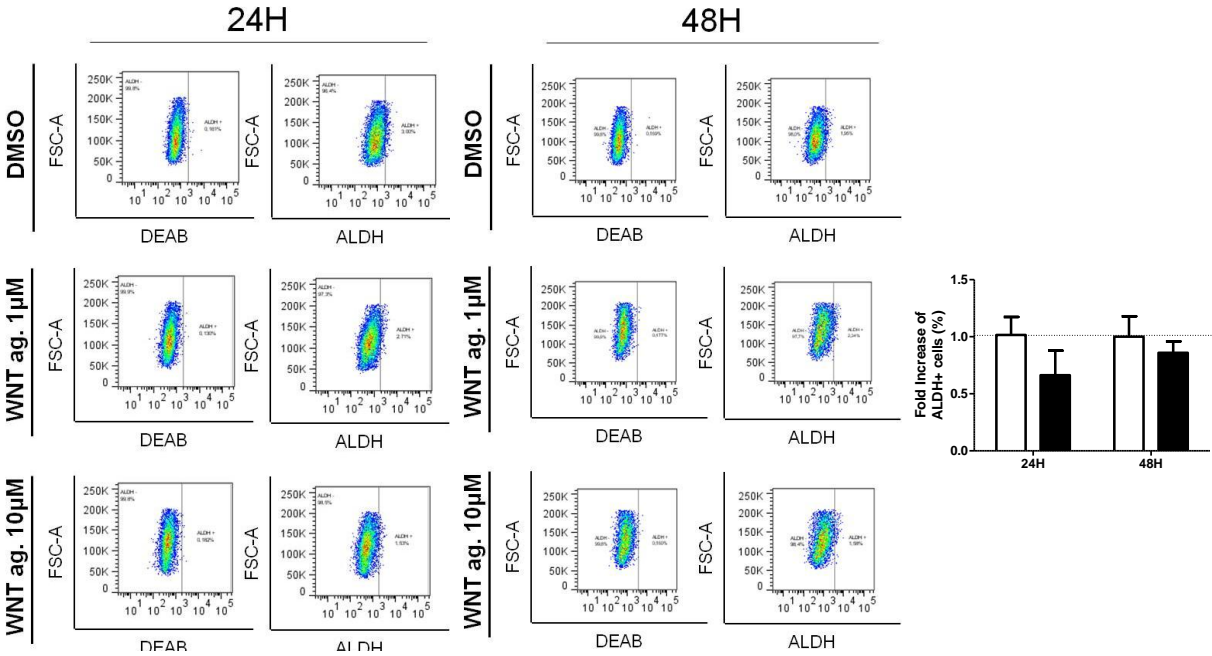

SUM149

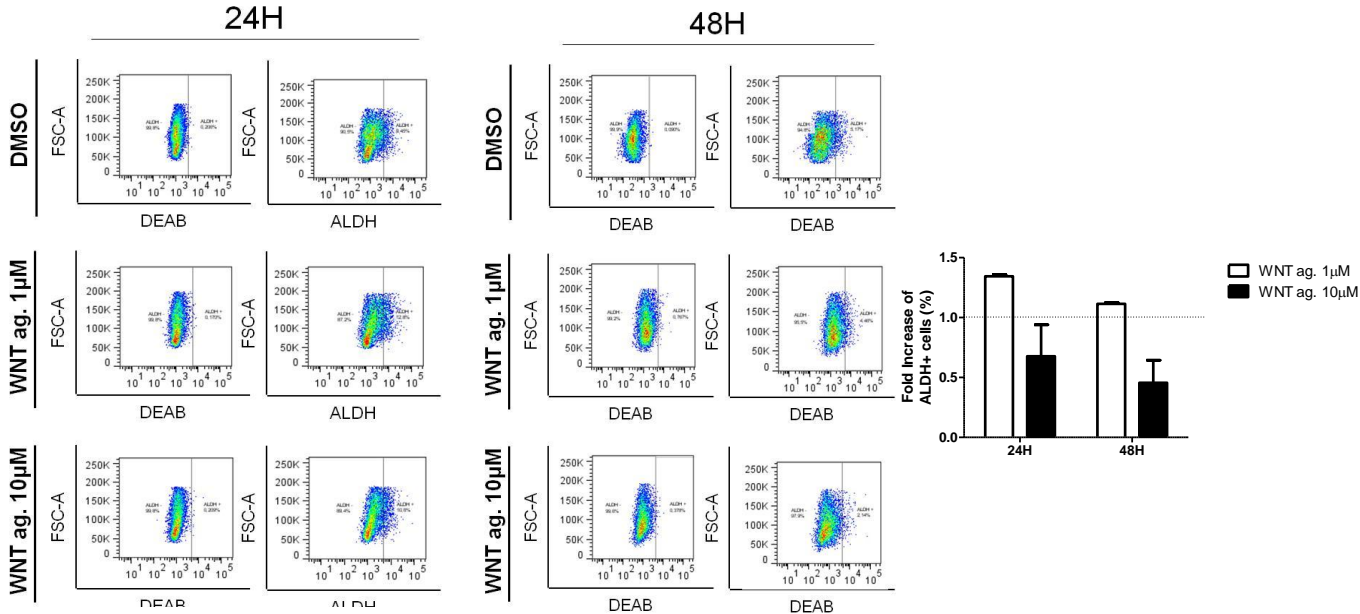

SUM159

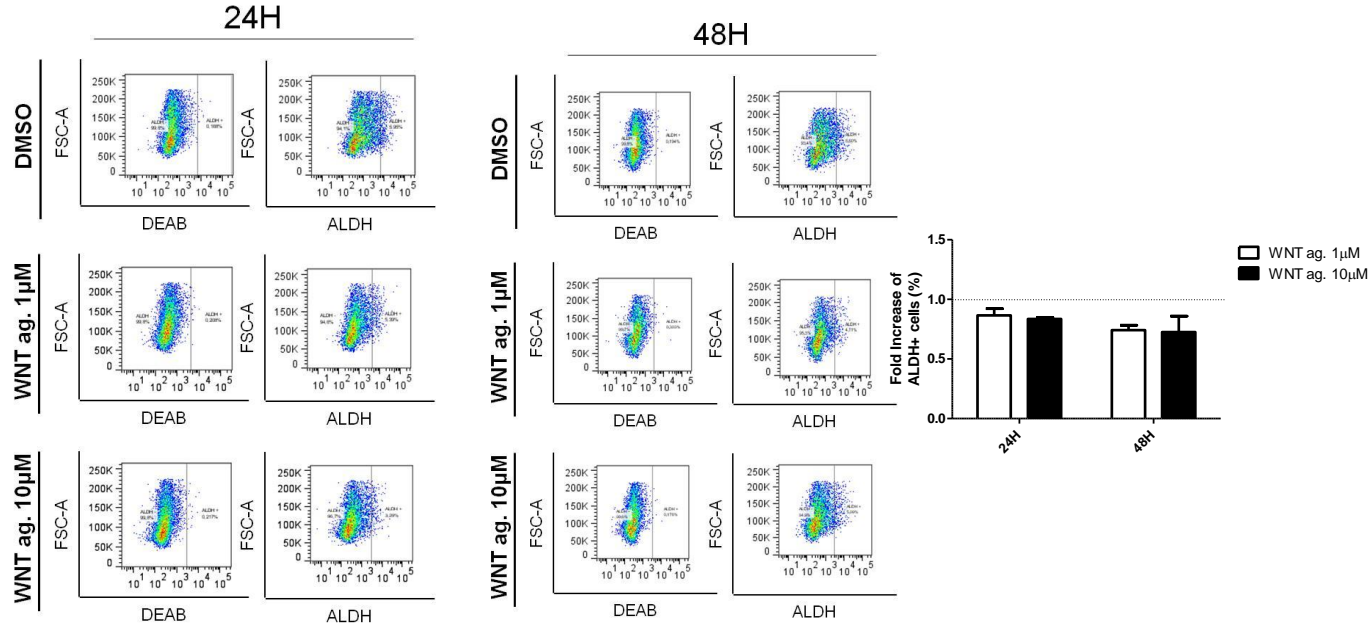

Supplement: Supplementary file 11 — Supplementary Figure S11 [file 41388_2019_700_MOESM11_ESM.pdf]

Supplementary Figure S12

MDAMB231

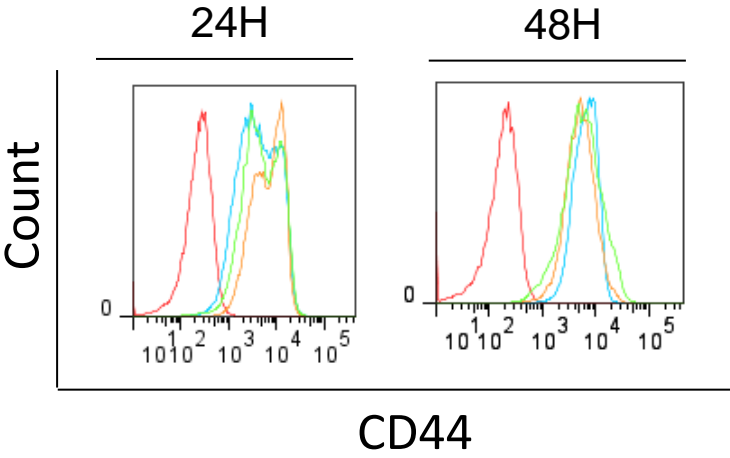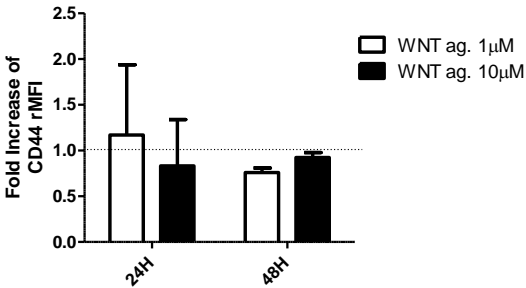

SUM149

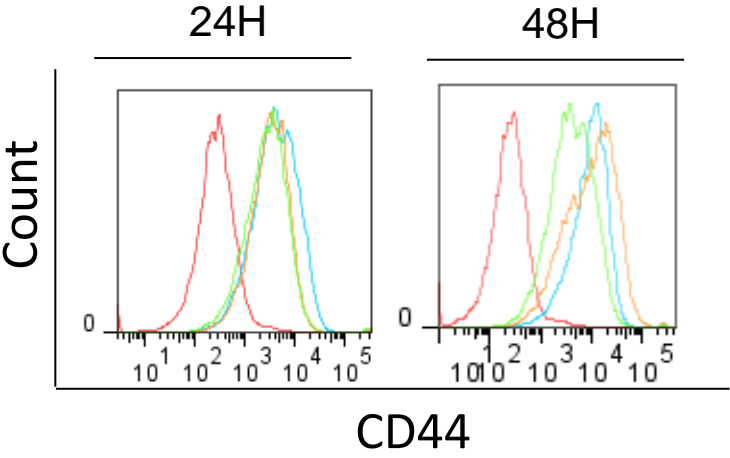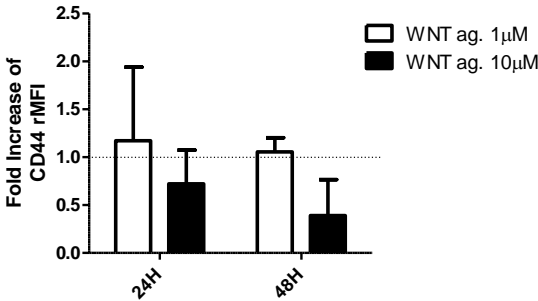

SUM159

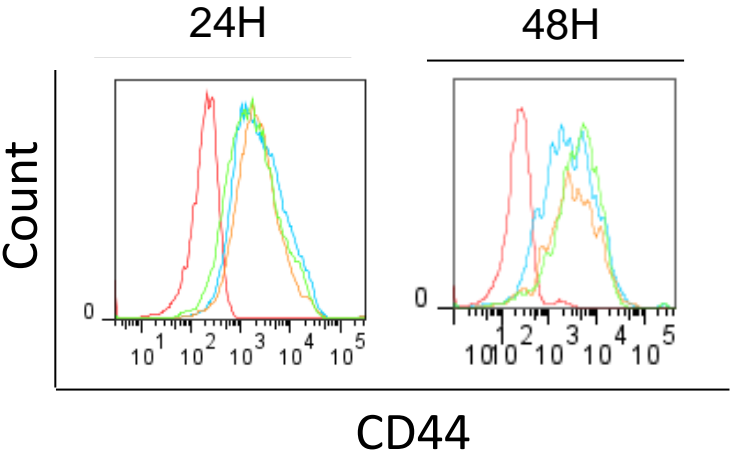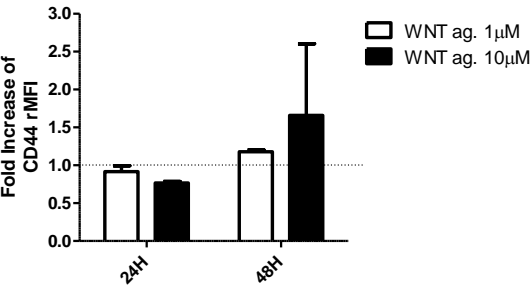

Ctrl Iso DMSO WNT ag.1µM WNT ag.10µM

Supplement: Supplementary file 12 — Supplementary Figure S12 [file 41388_2019_700_MOESM12_ESM.pdf]

Supplementary Figure S13

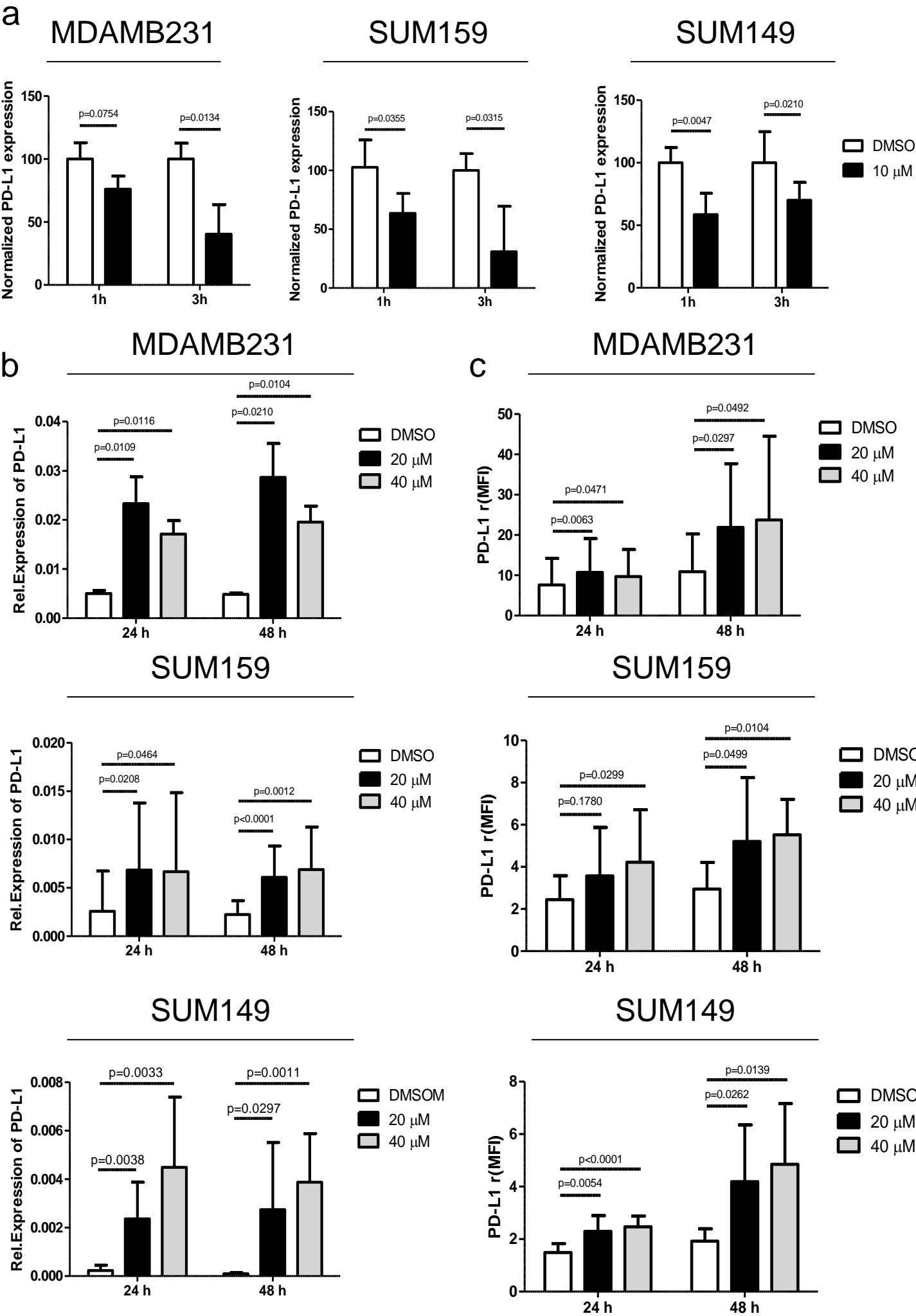

Supplement: Supplementary file 13 — Supplementary Figure S13 [file 41388_2019_700_MOESM13_ESM.pdf]

**MDAMB231**

24h

48h

Count

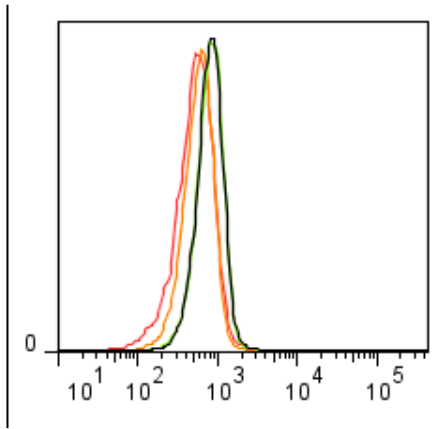

PD-L1

Count

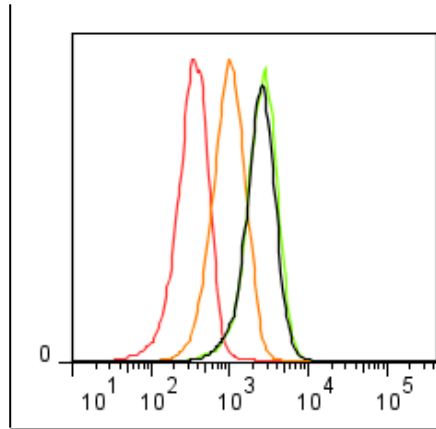

PD-L1

— 40μM  
— 20μM  
— DMSO  
— Ctrl Iso

**SUM149**

24h

48h

Count

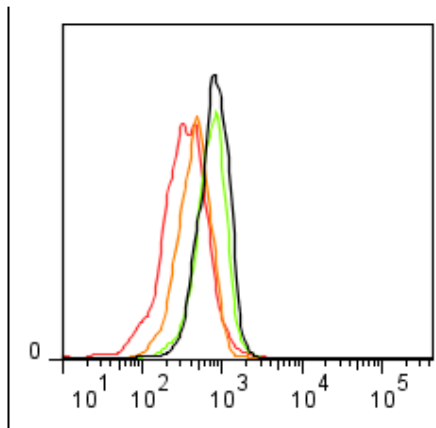

PD-L1

Count

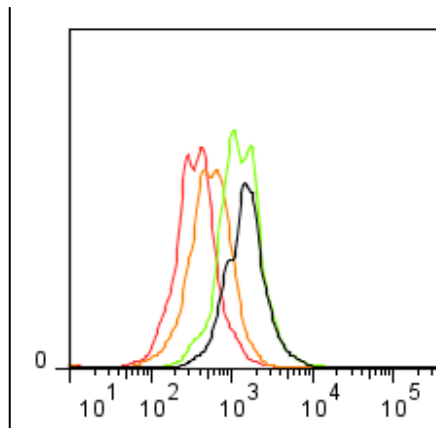

PD-L1

**SUM159**

**c**

Count

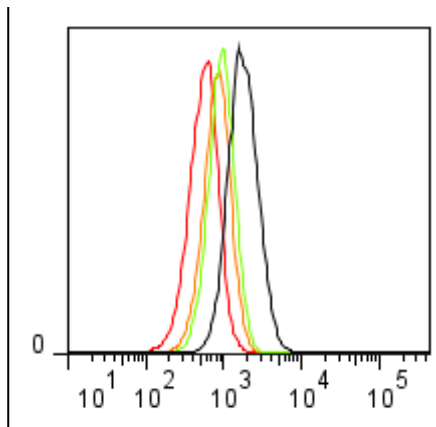

PD-L1

Count

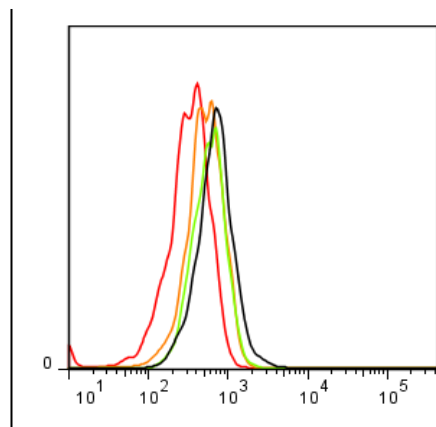

PD-L1

Supplement: Supplementary file 14 — Supplementary Figure S14 [file 41388_2019_700_MOESM14_ESM.pdf]

Supplementary Figure S15

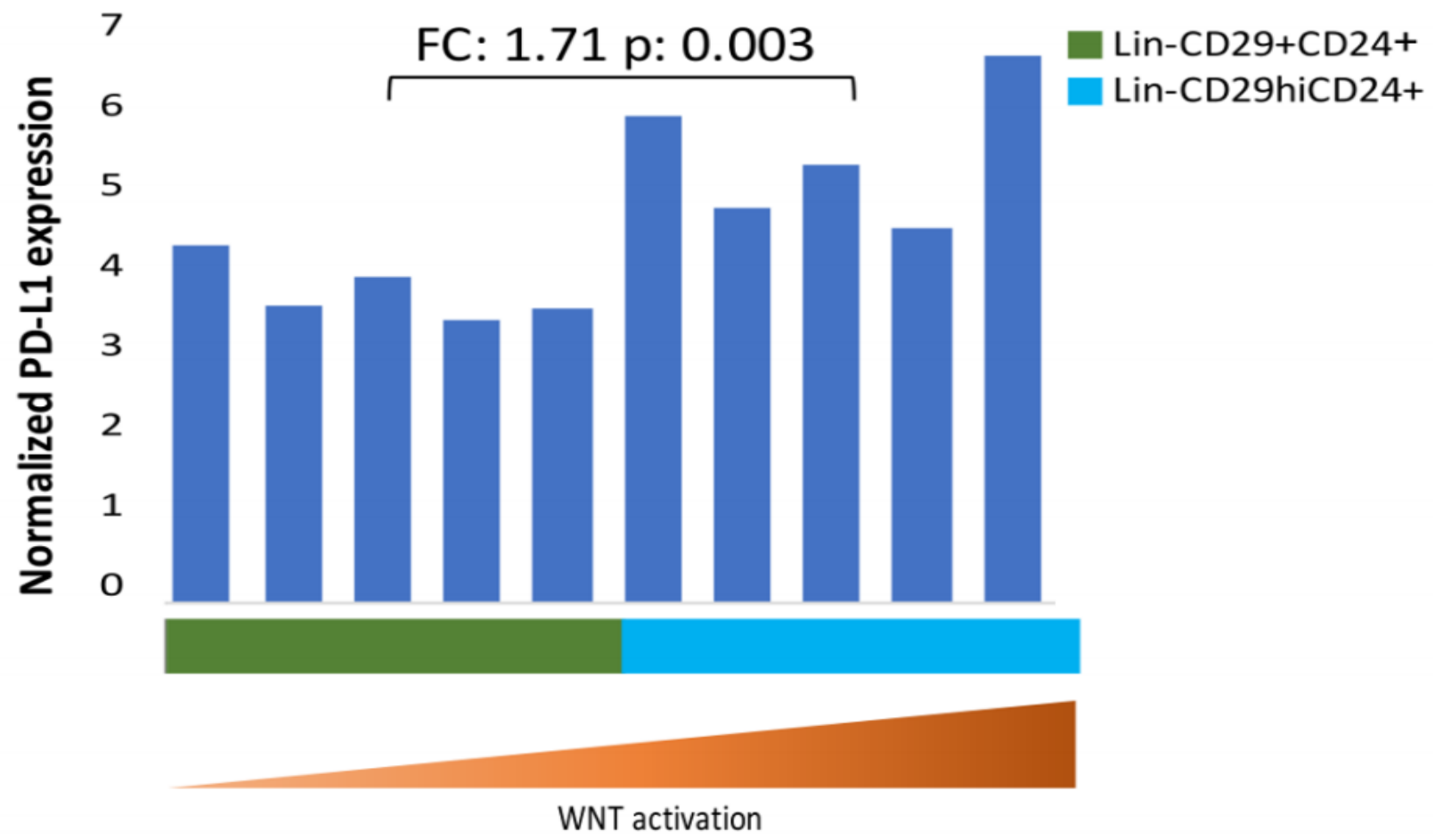

Supplement: Supplementary file 15 — Supplementary Figure S15 [file 41388_2019_700_MOESM15_ESM.pdf]
