# Supplementary material for: WNT signaling modulates PD-L1 expression in the stem cell compartment of triple-negative breast cancer
Source: Oncogene. 2019 Jan 31;38(21):4047–60. doi: 10.1038/s41388-019-0700-2 (PMC6755989; doi:10.1038/s41388-019-0700-2)
Supplement: Supplementary file 16 — Amended Supplementary Figure Legends not Highlighted [file 41388_2019_700_MOESM16_ESM.docx]

**Supplementary Figure S1. IHC staining of PD-L1 according to its expression levels in human TNBC samples.** Representative immunohistochemical staining for PD-L1 from two cases with high mRNA expression and two cases with low mRNA expression showing consistency between protein and transcript expression**.**

**Supplementary Figure S2.** Scatter plot of PD-L1 expression levels (z-score, x axis) and stemness score (SS) (z-score, y axis) in the different groups of TNBC. The correlation status was calculated by a Pearson correlation and p value for each group of tumors.

**Supplementary Figure S3. SS^High^/PD-L1^High^ samples show restricted stromal infiltration, significant immunological enrichment and high tumor cell content. a)** claudin-low score computed in the different TNBC subgroups classified according to SS score and PD-L1 expression. ESTIMATE algorithm analysis was performed to infer the fraction of stromal and immune cells in the bulk gene expression profiles across TNBC samples divided by SS and PD-L1 signals. Box and whisker plots shows: **b)** tumor purity (gene expression estimation) pattern among the Ital-Mex cohort (Dashed line represents mean value of all the cohort= 68%); **c)** stromal and **d**) the immune enrichment scores. **e)** Immunophenoscore (IPS) computed in each biological subgroup of Ital-Mex cohort. Kruskal–Wallis and Wilcoxon tests were computed, *, p < 0.05.

**Supplementary Figure S4. Representative dot plots showing PD-L1-expressing cells in ALDH+ and CD44**^High^ ***versus* ALDH-** and CD44^Low^ **cell counterpart in all TNBC cell lines. a)** The DEAB control sample was used as background fluorescence signal for the ALDEFLUOR™-stained cells. **b)** Representative plots of double stained cells showing PD-L1 expression in ALDH+ and ALDH- cells for each tested cell line. **c)** Representative plots of double stained cells showing PD-L1 expression in CD44^High^ and CD44^Low^ cells for each tested cell line.

**Supplementary Figure S5.** Representative plots showing the percentage of PD-L1^High^ *versus* PD-L1^Low^ cells in TNBC cell lines.

**Supplementary Figure S6.** Representative gating strategy showing the expression of PD-L1 compared with FMO within the gate of SCA-1-positive cells. FMO fluorescence was used to establish the cutoff for PD-L1-positive cells.

**Supplementary Figure S7. *In silico* analysis of WNT-related upstream and downstream gene expression alterations occurring in SS^High^/PD-L1^High^ TNBC cases and co-expression analysis of WNT signaling genes in TNBC Ital-Mex stratified according to PD-L1 expression levels. a)** Integrative WNT signaling pathway representation**.** Some pathway members are colored to represent the expression profiles of WNT-related genes in SS^High^ /PD-L1^High^ tumors. **b**) Gene expression analyses of altered WNT-related genes occurring in TNBC tumors (n=158) represented in heatmap plot; **c)** correlation matrix of WNT signaling genes in PD-L1^High^ (n=36) *versus* PD-L1^Low^ (n=55) TNBC cases of ITAL-MEX cohort computed by Pearson correlation. Purple areas indicate strong positive correlation between WNT-related pathway genes, whereas orange areas indicate negative correlation. Only genes with a significant correlation (p value < 0.05) were colored.

**Supplementary Figure S8**. Representative plots (left panels) showing the percentage of ALDH+, and bar plots (right panels) quantifying the fold increase of ALDH+ cells in MDAMB231, SUM149 and SUM159 cells upon treatment with the WNT-inhibitor XAV939 for 24 and 48h.

**Supplementary Figure S9**. FACS analyses showing CD44 expression (left panels) and bar plots (right panels) representing CD44 rMFI fold increase in MDAMB231, SUM149 and SUM159 cells upon treatment with the WNT-inhibitor XAV939 for 24 and 48h.

**Supplementary Figure S10.** Representative histograms showing the up-modulation of cell membrane-associated PD-L1 according to the dose of agonist (CAS 853220-52-7) tested and time of incubation in a) MDAMB231, b) SUM149 and c) SUM159

**Supplementary Figure S11.** Representative plots (left panels) showing the percentage of ALDH+ and bar plots (right panels) exhibiting the fold increase of ALDH+ cells MDAMB231, SUM149 and SUM159 cells upon treatment with the WNT-agonist CAS 853220-52-7 (WNT ag.) tested at 1 and 10µM for 24 and 48h.

**Supplementary Figure S12.** FACS analyses showing CD44 expression (left panels) and bar plots (right panels) exhibiting CD44 rMFI fold increase of MDAMB231, SUM149 and SUM159 cells upon treatment with the WNT-agonist CAS 853220-52-7 (WNT ag.) tested at 1 and 10µM for 24 and 48h in a) MDAMB231, b) SUM149 and c) SUM159

**Supplementary Figure S13. *In vitro* modulation of PD-L1 expression according to WNT inhibition (LGK-974) or activation (SKL2001)**. **a)** qRT-PCR analyses of PD-L1 transcript expression evaluated in MDAMB231, SUM159, and SUM149 cells treated with the WNT inhibitor LGK-974 (10 µM) or the diluent DMSO for 1 and 3 h. The values were normalized on PD-L1 expression in control samples. Column bars, mean ± SD (n=3). Significance was calculated by a two-tailed paired t-test. **b)** qRT-PCR analyses of PD-L1 transcript expression in MDAMB231, SUM159, and SUM149 cells treated with the selective WNT agonist SKL2001 at 20 and 40 µM or the diluent DMSO for 24 h and 48 h. Column bars, mean±SD (n=3). Significance was calculated by a two-tailed paired t-test. **c)** FACS analyses of PD-L1 expression in MDAMB231, SUM159, and SUM149 cells treated with the selective SKL2001 at 20 and 40 µM or the diluent DMSO for 24 h and 48 h. Columns, mean (n≥4) ±SD. Significance was calculated by a two-tailed paired t-test.

**Supplementary Figure S14.** Representative histograms showing the up-modulation of cell membrane-associated PD-L1 according to the dose of agonist (SKL2001) tested and time of incubation in a) MDAMB231, b) SUM149 and c) SUM159.

**Supplementary Figure S15. *In silico* analysis of the impact of PD-L1 expression on WNT activation in murine stem cells derived from spontaneous Apc1572T/+ mammary lesions sorted for Lin-CD29 and CD24 levels.** Bar plots summarizing the expression values of PD-L1 in stem (Lin-CD29hiCD24+) *versus* differentiated/bulk (Lin-CD29+CD24+) *ex vivo* tumor cell suspensions of different Apc1572T/+ TNBC samples.
